# Novel Compounds Featuring a Thiophene Carboxamide Scaffold: Synthesis, Characterization and Antiproliferative Evaluation

**DOI:** 10.3390/ijms26146823

**Published:** 2025-07-16

**Authors:** Bogdan-Ionuț Mara, Alexandra Mioc, Livia-Nicoleta Deveseleanu-Corici, Codruța Șoica, Liliana Cseh

**Affiliations:** 1 Coriolan Dragulescu Institute of Chemistry, Romanian Academy, Bv. M. Viteazu, No. 24, 300223 Timișoara, Romania; 2 Faculty of Pharmacy, Victor Babes University of Medicine and Pharmacy, Eftimie Murgu Square, No. 2, 300041 Timișoara, Romania; 3 Research Center for Experimental Pharmacology and Drug Design, Victor Babes University of Medicine and Pharmacy, Eftimie Murgu Square, No. 2, 300041 Timișoara, Romania

**Keywords:** thiophene carboxamide derivatives, toxicity assay, anticancer

## Abstract

Thiophene derivatives are particularly attractive for application in drug development for their versatile pharmacological properties. We synthesized a series of four compounds with thiophene carboxamide as a scaffold. The structures were established based on HR-MS and 1D- and 2D-NMR. The purity of the compounds was established to be greater than 92% by thin-layer chromatography and NMR. The cytotoxic effects of the newly synthesized compounds were evaluated against the normal HaCaT cell line and A375, HT-29, and MCF-7 cancer cell lines. The cytotoxic assessment revealed that two compounds exhibit a significant cytotoxic effect on all cancer cell lines. To investigate their potential underlying mechanisms of action, several tests were performed: immunofluorescence imaging, caspase-3/7 assay, mitochondrial membrane potential (JC-1) assay, and 2′,7′–dichlorofluorescein diacetate (DCFDA) assay. **MB-D2** proved to be the most cytotoxic and effective in terms of caspase 3/7 activation, mitochondrial depolarization and decrease in ROS production; these effects did not occur in normal HaCaT cells, revealing that **MB-D2** has a high selectivity against A375 cancer cells.

## 1. Introduction

Heterocyclic structures are widely distributed in nature and possess great synthetic applicability and biological activity, which have led medicinal chemists to implement new strategies towards the discovery of novel drugs [1]. Thiophene is a remarkable heterocycle found in an overwhelming number of medicinal compounds possessing versatile pharmacological properties [1]. The substitution of a ring carbon atom by sulfur in thiophene results in significant changes to the electronic distribution and molecular geometry due to differences in electronegativity, electron configuration, and the presence of lone pairs [2]. These modifications enhance the reactivity and biological potential of thiophene derivatives. Biologically, thiophene is incorporated in several cofactors, sugars, and nucleic acids, and it plays regulatory roles in processes such as tRNA sulfuration [2]. Moreover, thiophene-based molecules are used as key intermediates in various industries and therapeutic areas [3].

Cancer remains a major health burden globally, largely due to its high metastatic potential and the development of resistance to conventional therapies such as radiotherapy and chemotherapy [4,5]. Traditional chemotherapeutic agents are often limited by low solubility, poor bioavailability, systemic toxicity, and acquired resistance [6,7]. Consequently, the development of novel anticancer agents is a continuous focus, with thiophene derivatives gaining substantial interest in recent decades.

The aromaticity and planarity of the thiophene ring enhance receptor binding, while its chemical structure allows for functionalization that can improve selectivity and potency. These properties have made thiophene derivatives attractive candidates for targeting cancer-related proteins such as kinases and apoptosis modulators [7]. The electron delocalization of sulfur within the π-system contributes to thiophene’s behavior as a reactive benzene analog, reinforcing its value as a pharmacophore with diverse biological effects [8].

Notably, 2-bromo-5-substituted thiophenes have demonstrated potent cytotoxicity. For example, 2-bromo-5-(2-methylphenyl)thiophene (BMPT) showed selective anticancer activity against HepG2 and Caco-2 cell lines via caspase-3/8/9 activation and Bcl-2 suppression, with EC_50_ values in the low micromolar range [9]. Furthermore, structural modifications of this core, such as the introduction of an oxime functional group with a z configuration, led to enhanced activity, with submicromolar IC_50_ values against MCF-7 cells (e.g., 0.28 μM) [10].

In parallel, thiophene carboxamide scaffolds have emerged as promising anticancer agents. Members of the PAN-90806 family were identified as potent VEGFR-2 inhibitors, inducing apoptosis and disturbing redox homeostasis with nanomolar-range IC_50_ values [11]. Other analogs, such as JCI-20679, have displayed mitochondrial complex I inhibition, yielding in vitro and in vivo antiproliferative effects [12]. Additionally, thiophene-2-carboxamides bearing aryl substituents demonstrated cytotoxicity in breast, liver, and leukemia cell lines, potentially via PTP1B inhibition [13].

Early studies by Shams et al. reported cytotoxic effects of several thiophene derivatives across central nervous system, breast, and non-small cell lung cancer cell lines, with one compound displaying consistent activity across all models [14]. More recently, additional derivatives have been screened against breast cancer cells, showing strong antiproliferative effects [15].

Building upon these findings, the current study focuses on the synthesis and biological evaluation of four novel compounds (**MB-D1** to **MB-D4**) incorporating a thiophene carboxamide core functionalized with bromine and imide or amide groups. These structural features were selected based on literature precedents supporting their anticancer potential. The compounds were tested against one normal cell line (HaCaT) and three cancer cell lines: A375 (melanoma), HT-29 (colorectal cancer), and MCF-7 (breast cancer), aiming to assess their cytotoxicity and therapeutic relevance.

## 2. Results and Discussion

### 2.1. Chemistry

In the present study, we investigated the antiproliferative activity of two amides (5-bromo-N-(thiazol-2-yl)thiopene-2-carboxamide-**MB-D1** and 5-bromothiophen-2-yl)(morpholino)methanone-**MB-D3**) and two imides (5-bromo-N-(5-bromothiophene-2-carbonyl)-N-(1,5-dimethyl-3-oxo-2-phenyl-2,3-dihydro-1H-pyrazol-4-yl)thiophene-2-carboxamide-**MB-D2** and dimethyl 4,4′- ((((1,5- dimethyl-3-oxo-2-phenyl-2,3-dihydro-1H-pyrazol-4-yl)azanediyl)bis(carbonyl))bis(thiophene-5,2-diyl))dibenzoate-**MB-D4**), which were synthesized using 5-bromo-2-thiophenecarboxylic acid as a building block.

**Scheme 1:**
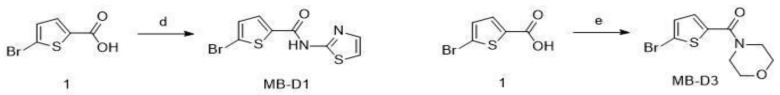
Synthesis of **MB-D1** and **MB-D3**. Reagents: (d) 2-aminothiazole, DCC, DMAP, and DCM at r.t.; (e) morpholine, DCC, DMAP, and DCM at r.t.

The **MB-D1** and **MB-D3** amides were prepared from the building-block unit with thiazol-2-amine and morpholine, respectively, using the procedure described in [16], with slight modifications; thus, 4-dimethyl aminopyridine was used as an acyl transfer reagent instead of triethylamine, and N,N-dicyclohexyl-carbodiimide was used a as a dehydrating agent (Scheme 1).

**Scheme 2:**
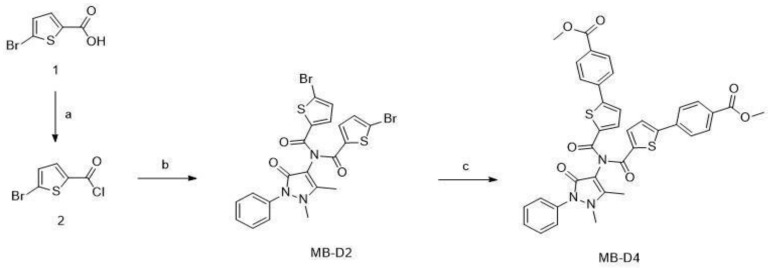
Synthesis of **MB-D2** and **MB-D4**. Reagents: (a) SOCl_2_ and DCM; (b) 4-aminoantipyrine, TEA, and DCM; (c) (4-(methoxycarbonyl)phenyl) boronic acid, Pd(PPh_3_)_4_, Cs_2_CO_3_, and 1,4-dioxane at 90 °C.

The **MB-D2** imide was prepared using the building block and thionyl chloride in order to obtain 5-bromothiophene-2-carbonyl chloride. The crude 5-bromothiophene-2-carbonyl chloride was reacted with 4-aminoantipyrine to obtain **MB-D2**. **MB-D2** was modified by Suzuki–Miyaura reaction to obtain **MB-D4** using (4-(methoxycarbonyl)phenyl) boronic acid in the presence of a palladium catalyst (Scheme 2).

High-resolution MS analysis was useful for confirming molecular formulas for the newly synthesized compounds. All the compounds showed high signal intensities in positive-ion mode with an error of less than 1 ppm for the molecular ion peak [M+H]^+^. **MB-D1**, **MB-D2**, and **MB-D3** produced distinct molecular ion clusters due to the combinations of bromine isotopes (79 and 81). Therefore, **MB-D1** afforded two molecular ions at m/z 288.91010 [C_8_H_5_^79^BrN_2_OS_2_+H]^+^ and 290.90787 [C_8_H_5_^81^BrN_2_OS_2_+H]^+^ with a ratio of 1:1 (Figures S1 and S2); **MB-D2** afforded three molecular ions at *m*/*z* 579.89966 [C_21_H_15_^79^Br_2_N_3_O_3_S_2_+H]^+^, 581.89740 [C_21_H_15_^79^Br^81^BrN_3_O_3_S_2_^+^]^+^, and 583.89496 [C_21_H_15_^81^Br_2_N_3_O_3_S_2_+H]^+^ with a ratio of 1:2:1 (Figures S3 and S4); **MB-D3** showed the cluster of ion peaks at *m*/*z* 275.96890 [C_9_H_10_^79^BrNO_2_S +H]^+^ and 277.96671 [C_8_H_10_^81^BrNO_2_S+H]^+^ with a ratio 1:1 (Figures S5 and S6) and **MB-D4** showed the molecular ion peak at *m*/*z* 692.15204 [C_37_H_29_N_3_O_7_S_2_ +H]^+^ (Figures S7 and S8).

### 2.2. Biological Evaluation

#### 2.2.1. Evaluation of the Cytotoxic Effect

**Figure 1:**
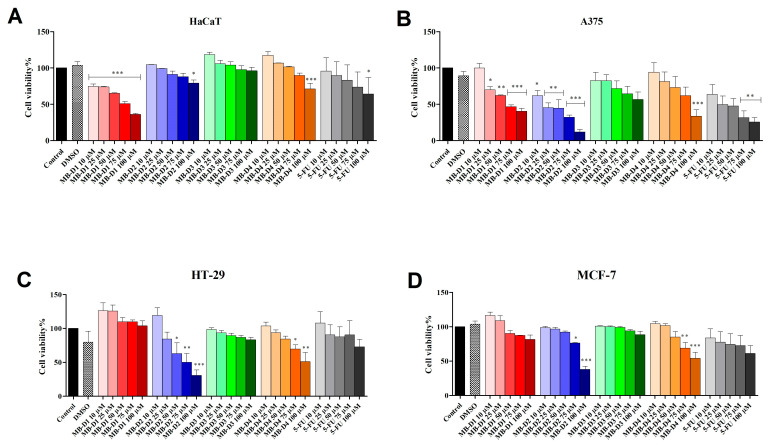
Cell viability after 48 h treatment of HaCaT (**A**), A375 (**B**), HT-29 (**C**), and MCF-7 (**D**) cells with 5-FU, **MB-D1**, **MB-D2**, **MB-D3**, and **MB-D4** (10, 25, 50, 75, and 100 μM). The results are expressed as viability percentages in comparison with the control group, considering 100% (* *p* < 0.05, ** *p* < 0.01 and *** *p* < 0.001). The data represent the mean values ± SD; n = 3 per group.

Following a 48 h treatment period, the cytotoxic effects of the **MB-D1**, **MB-D2**, **MB-D3**, and **MB-D4** compounds (10, 25, 50, 75, and 100 μΜ) on HaCat, MCF-7, HT-29, and A375 cells was evaluated by employing an Alamar blue assay (Figure 1). In HaCat cell lines **MB-D3** did not exert significant cytotoxicity, even at higher concentrations (100 μΜ); however, **MB-D1**, **MB-D**2 and **MB-D4** significantly decreased cell viability as follows: for **MB-D1**, 36.02% ± 2.7 (100 μΜ) and 50.79% ± 5.2 (75 μΜ); for **MB-D2**, 79.23% ± 7.39 (only at 100 μΜ); and for **MB-D4**, 71.05% ± 2.44 (100 μΜ). At 100 μΜ, the positive control, 5-FU, decreased cell viability at 64.19% ± 21.42. In terms of anticancer activity, **MB-D3** did not exert any significant effects against any of the used cancer cell lines, even at higher concentrations (100 and 75 μΜ). **MB-D1**, **MB-D2** and **MB-D4** exhibited promising antiproliferative effects against the MCF-7, HT-29 and A375 cell lines at higher concentrations of 100, 75 and 50 μΜ. Against the MCF-7 cell line, the most active compound proved to be **MB-D2**, exhibiting 38.93% ± 8.19 cell viability at 100 μΜ and 76.18% ± 1.4 cell viability at 50 μΜ, followed by 53.98% ± 19.46 for **MB-D4** at 100 μΜ and 68.75% ± 18.3 cell viability at 50 μΜ. Upon testing on HT-29 colon cancer cell lines, **MB-D2** and **MB-D4** exhibited promising cytotoxic effects, reducing cell viability at 100 and 75 μΜ (30.6% ± 18.4, 50.04% ± 22.8, 69.28% ± 13.65, and 51% ± 23.2 compared to the control (100%)). Against A375 melanoma cells, **MB-D2** proved the most effective, significantly reducing the cell viability on the higher tested concentrations of 100 μΜ (11.74% ± 6.061) and 75 μΜ (31.96% ± 5.1), followed by **MB-D4** at 100 μΜ (33.42% ± 8.8) and **MB-D1** at 100 μΜ (40.31% ± 7.9) and 75 μΜ (46.40% ± 4.2) vs. rgw control (100%). The positive 5-FU control decreased cell viability as follows: 25.58% ± 11.9 at 100 μΜ, 31.45% ± 18.6 at 75 μΜ, and 47.62% ± 19.6 at 50 μΜ. Based on the obtained results, **MB-D1**, **MB-D2**, and **MB-D4** were considered for further testing. Similar cytotoxic effects of various thiophene derivatives were reported by Shah et al. [17], who revealed that a compound with electron-withdrawing groups (*p*-Br) on benzylidene portion exhibited significant anticancer activity against a human lung cancer cell line (A-549) at a dose of 10^−4^ M. Another thiophene derivative, F8 (methyl 5-[(dimethylamino)carbonyl]-4-methyl-2-[(3-phenyl-2-propynoyl)amino]-3-thiophenecarboxylate), was shown to display anticancer activity against lymphoma (CCRF-CEM), leukemia (JURKAT and HL-60), breast adenocarcinoma (MDA-MB-231), pancreatic carcinoma (PANC-1), and malignant melanoma (A375) cell lines, with the 50% cytotoxic concentration (CC_50_) in the μM range [18].

The calculated IC_50_ values (μM) after 48 h treatment with **MB-D1**, **MB-D2**, **MB-D3**, and **MB-D4** are presented in Table 1.

#### 2.2.2. Evaluation of Cell Morphology

**Figure 2:**
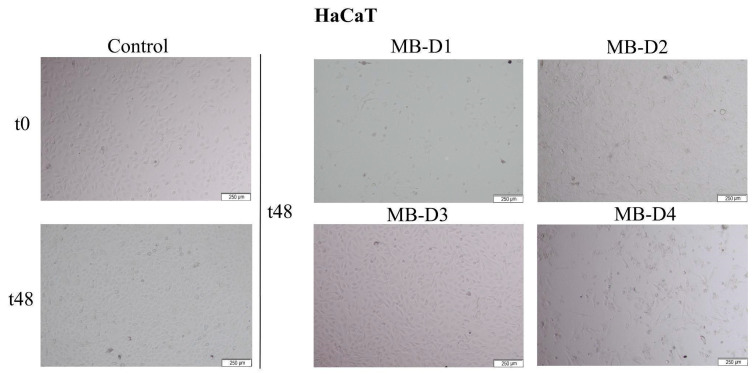
Morphological changes in HaCaT cells after 48 h treatment with **MB-D1**, **MB-D2**, **MB-D3**, and **MB-D4** (100 μM); the scale bar is 250 μm.

**Figure 3:**
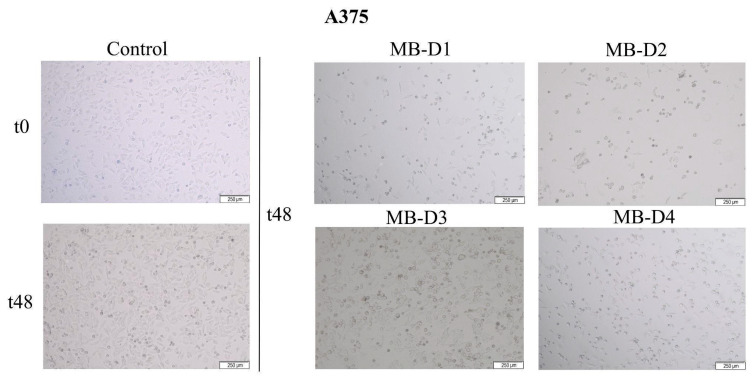
Morphological changes in A375 cells after 48 h treatment with **MB-D1**, **MB-D2**, **MB-D3**, and **MB-D4** (100 μM); the scale bar is 250 μm.

**Figure 4:**
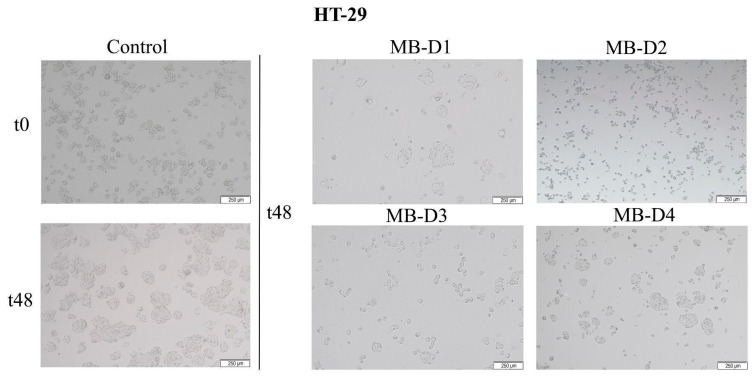
Morphological changes in HT-29 cells after 48 h treatment with **MB-D1**, **MB-D2**, **MB-D3**, and **MB-D4** (100 μM); the scale bar is 250 μm.

**Figure 5:**
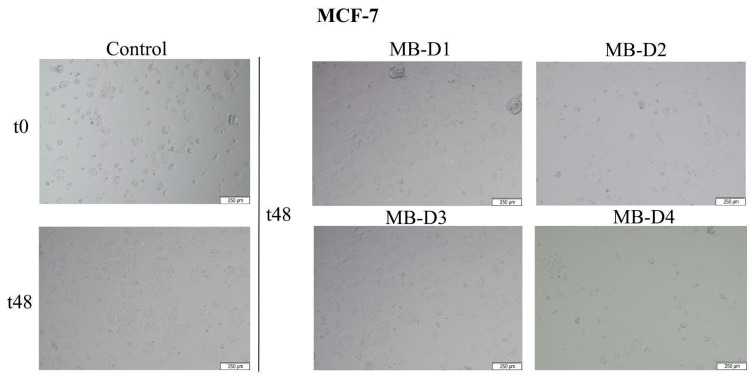
Morphological changes in MCF-7 cells after 48 h treatment with **MB-D1**, **MB-D2**, **MB-D3**, and **MB-D4** (100 μM); the scale bar is 250 μm.

No significant morphological changes were observed regarding confluence or aspect between the HaCaT cells treated with **MB-D3** and the control cells. Treatment with **MB-D2** resulted in an increased number of detached cells. However, treatment with **MB-D1** and **MB-D4** decreased the overall number of cells, rendering the remaining cells detached and round (Figure 2). Treatment with **MB-D1**, **MB-D2**, **MB-D3**, and **MB-D4** caused the A375 and HT-29 cells to detach, become more rounded, and become less confluent to varying degrees; these treatments clearly induced noticeable changes in the morphology of these cells (Figure 3 and Figure 4). In contrast, in the MCF-7 cell line, only treatment with **MB-D2** and **MB-D4** induced similar changes, as described above (Figure 5).

During apoptosis, distinct morphological changes occur, such as nuclear fragmentation, chromatin condensation, cell shrinkage, disassembly/cleavage of the cell cytoskeleton, membrane blebbing, and the formation of apoptotic bodies [19]. In order to observe the exact changes produced by the 48 h treatment with **MB-D1**, **MB-D2** and **MB-D4**, the cells’ nuclei were stained using Hoechst dye, and their cytoskeletons were labelled with F-actin. Moreover, activated caspase-3/7 was detected using a Caspase-3/7 Green Detection Reagent. Based on the cell viability results and given that compound MB-D3 had no effect on the cell viability of A375, HT-29, or MCF-7, we decided to exclude this compound from subsequent experiments.

**Figure 6:**
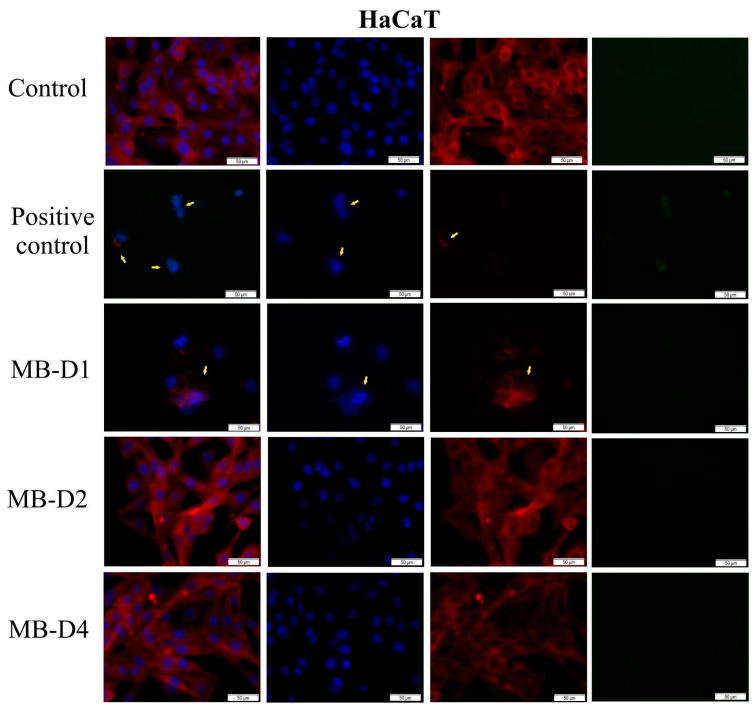
The effect of 48 h treatment with **MB-D1**, **MB-D2**, and **MB-D4** (IC_50_/100 μM) on HaCaT cell nuclei (blue—Hoechst staining), cytoskeletons (F-actin—red staining), and caspase 3/7 activation (green staining). Staurosporine (5 μM) was used as a positive control for apoptotic cell death. The scale bar is 50 μm.

**Figure 7:**
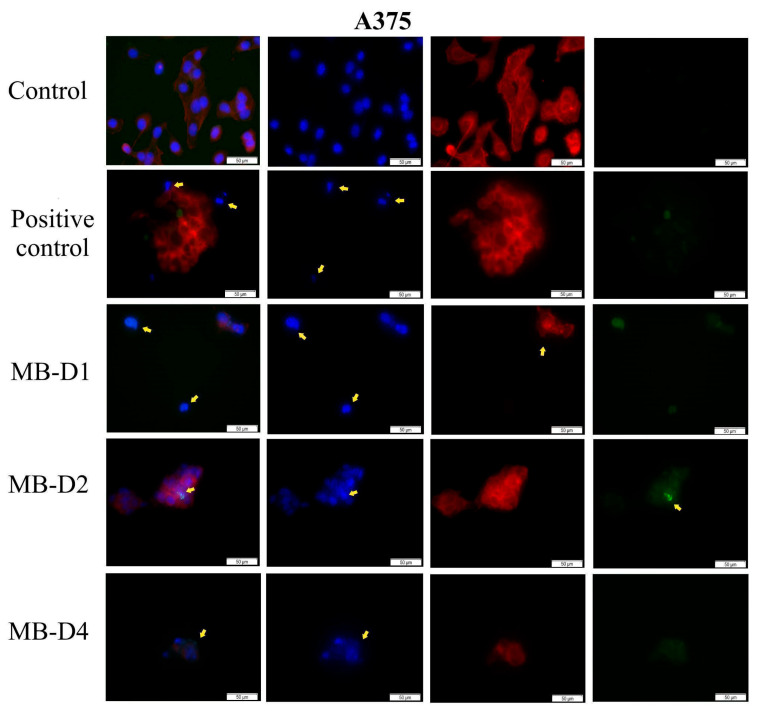
The effect of 48 h treatment with **MB-D1**, **MB-D2**, and **MB-D4** (IC_50_/100 μM) on A375 cell nuclei (blue—Hoechst staining), cytoskeleton (F-actin—red staining), and caspase 3/7 activation (green staining). Staurosporine (5 μM) was used as a positive control for apoptotic cell death. The scale bar is 50 μm.

**Figure 8:**
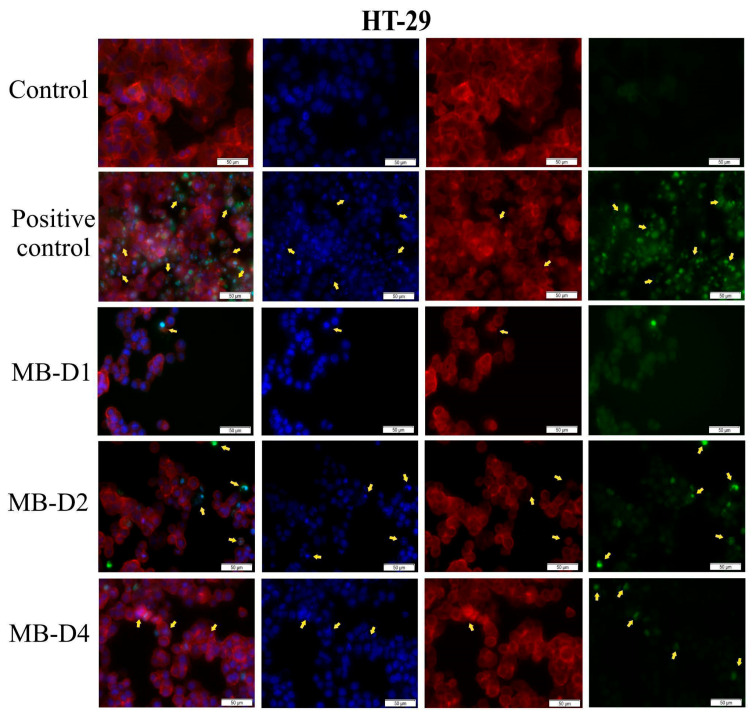
The effect of 48 h treatment with **MB-D1**, **MB-D2**, and **MB-D4** (IC_50_/100 μM) on HT-29 cell nuclei (blue—Hoechst staining), cytoskeleton (F-actin—red staining), and caspase 3/7 activation (green staining). Staurosporine (5 μM) was used as a positive control for apoptotic cell death. The scale bar is 50 μm.

**Figure 9:**
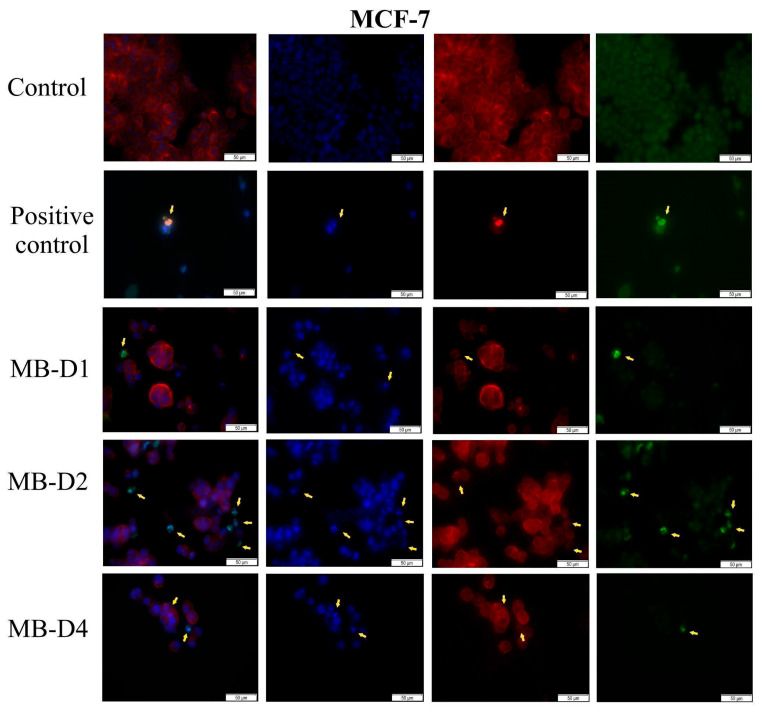
The effect of 48 h treatment with **MB-D1**, **MB-D2**, and **MB-D4** (IC_50_/100 μM) on MCF-7 cell nuclei (blue—Hoechst staining), cytoskeleton (F-actin—red staining), and caspase 3/7 activation (green staining). Staurosporine (5 μM) was used as a positive control for apoptotic cell death. The scale bar is 50 μm.

The specific signs of apoptosis were observed in HaCaT cells treated only with **MB-D1** (Figure 6). In contrast, A375, HT-29, and MCF-7 cells showed specific signs of apoptosis when treated with all the compounds used in this study (Figure 7, Figure 8 and Figure 9). Caspase 3/7 activation was observed in all the cell lines treated with the compounds, with the exception of the HaCaT cell line (Figure 6, Figure 7, Figure 8 and Figure 9).

In our study, the morphological assessment using immunofluorescence revealed that treatment of A375, HT-29, and MCF-7 cells with the newly synthesized compounds (**MB-D1**, **MB-D2**, and **MB-D4**) induced morphological alterations characteristic of apoptosis. Furthermore, treatment with the compounds enhanced the signal intensity of cancer cells stained with caspase-3/7 antibody. Taken together, these results strongly suggest the activation of the apoptotic pathway. Apoptosis, or programmed cell death, can be triggered through two pathways: the extrinsic and the intrinsic pathways. For the activation of the extrinsic pathway, death ligands, such as tumor necrosis factor (TNF)-α, Fas ligand (FasL), or TNF-related apoptosis-inducing ligand (TRAIL), have to bind to the corresponding death receptors, which consecutively lead to the activation of caspase 8 and 10. In turn, the activated caspase 8/10 leads to the activation of caspase 3/6/7 and, ultimately, to the induction of apoptosis [20,21]. In contrast, the intrinsic pathway, also called the mitochondrially mediated apoptotic pathway, is regulated by the Bcl-2 protein family. The activation of the intrinsic pathways occurs as a response to extracellular and intracellular stressors such as oxidative stress, DNA damage, irradiation, or disruption of signals that normally promote cell survival. These stressors lead to the activation and oligomerization of the proapoptotic Bax and Bak proteins at the level of the mitochondrial outer membrane, consecutively promoting a defining event, i.e., mitochondrial outer-membrane permeabilization (MOMP). Following MOMP, cytochrome C is released from mitochondria and successively leads to the formation of the apoptosome and caspase 9 activation; this activation leads to the final activation of caspase 3/6/7 and, ultimately, to apoptosis [20,21].

#### 2.2.3. Effects of **MB-D1**, **MB-D2**, and **MB-D4** Derivatives on Caspase-3/-7 Production

**Figure 10:**
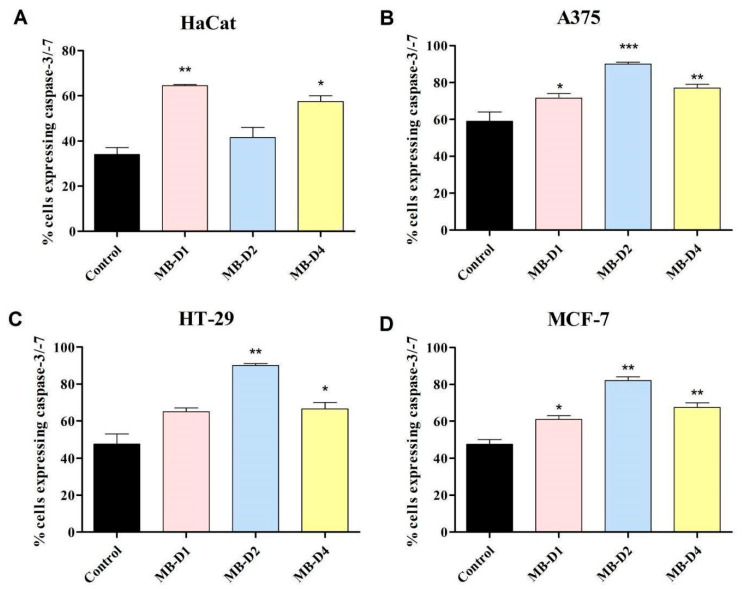
The activation of caspase 3/7 in HaCaT (**A**), A375 (**B**), HT-29 (**C**), and MCF-7 (**D**) cell lines after 48 h treatment with **MB-D1**, **MB-D2**, and **MB-D4** (IC_50_/100 μM). The experiments were performed in triplicate, and results were calculated as mean ± SD; n = 3 per group. * *p* < 0.05, ** *p* < 0.01, and *** *p* < 0.001.

In the next step, to quantitatively confirm caspase-3/7 activation and demonstrate the compounds’ ability to induce apoptotic cell death, HaCaT, A375, HT-29, and MCF-7 cells were treated with **MB-D1**, **MB-D2**, and **MB-D4** (IC_50_/100 μM) and directly assessed using the caspase-3/7 Green Detection Reagent kit and an automated fluorescent cell counter. The results presented in Figure 10 show that in A375 and MCF-7, all the compounds were able to significantly increase caspase 3/7 activation vs. the control. **MB-D1** did not increase caspase 3/7 in HT-29 cells. However, both **MB-D1** and **MB-D4** significantly increased caspase 3/7 activation in normal HaCaT cells. Treatment with **MB-D2** did not increase caspase 3/7 activation in HaCaT cells; this result highlights the selective cytotoxic effect of this compound and its ability to induce apoptosis in cancer cells (Figure 10).

#### 2.2.4. Effects if **MB-D1**, **MB-D2**, and **MB-D4** Derivatives on Mitochondrial Membrane Potential

**Figure 11:**
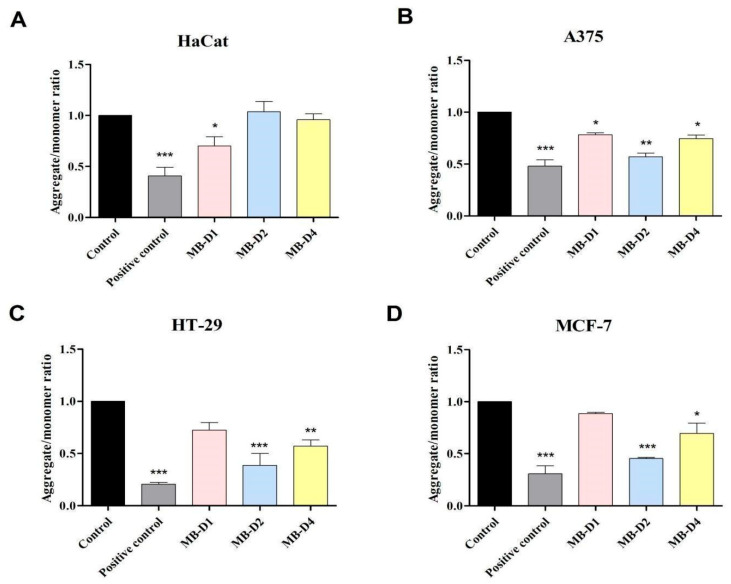
The effect of 48 h treatment with **MB-D1**, **MB-D2**, and **MB-D4** (IC_50_/100 μM) on the mitochondrial membrane in HaCaT (**A**), A375 (**B**), HT-29 (**C**), and MCF-7 (**D**) cell lines, expressed as the JC-1 aggregate/monomer ratio. The results are expressed as mean  ±  SD; n = 3 per group. * *p* < 0.05, ** *p* < 0.01, and *** *p* < 0.001.

Over the last two decades, mitochondria have emerged as significant pharmacological targets in cancer therapy due to their essential role in cell proliferation and death. In healthy cells, mitochondria are known to maintain an electrochemical gradient, i.e., a mitochondrial transmembrane potential (Δψm), across the inner mitochondrial membrane (IMM), which is essential for ATP production, protein import, and lipid biogenesis [22]. As revealed by numerous studies, the loss of Δψm and the depolarization of mitochondria is associated with apoptosis and cell death [23,24]. As discussed in the previous section, during the activation of the intrinsic apoptotic pathway, Bax and Bak proteins promote MOMP, thereby inducing cytochrome C release and apoptosis; during these events, mitochondrial loss of Δψm may occur [25,26]. In order to determine whether the proapoptotic effect and activation of caspase 3/7 observed in A375, HT-29, and MCF-7 cells after treatment with **MB-D1**, **MB-D2**, and **MB-D4** occurred through the activation of the intrinsic or extrinsic apoptotic pathway, we evaluated the mitochondrial membrane potential using a JC-1 assay. In normal conditions and in healthy mitochondria, the monomer form of the JC-1 cationic dye (green fluorescence) enters the mitochondria and forms JC-1 aggregates (red fluorescence). In contrast, in dysfunctional mitochondria, due to mitochondrial membrane depolarization disruptions, the JC-1 cannot enter the mitochondria, and the ratio between the aggregate/monomer (red/green form) decreases. Reflecting the mitochondrial membrane potential (MMP), the aggregate/monomer ratio was detected in each cell line treated with **MB-D1**, **MB-D2**, and **MB-D4** (IC_50_/100 μM); FCCP was used as a positive control for MMP depolarization. The results are presented in Figure 11. Specifically, compounds **MB-D2** and **MB-D4** did not exert any statistically significant effect on the HaCaT cell line vs. the control. **MB-D1** decreased the aggregate/monomer ratio vs. the control (0.702 ± 0.11 vs. 1), indicating MMP depolarization. A similar effect was observed in the A375 cell line after treatment with **MB-D1**, **MB-D2**, and **MB-D4** as follows: 0.783 ± 0.02, 0.5695 ± 0.05, and 0.7444 ± 0.04 vs. 1. In the HT-29 cell line, **MB-D2** and **MB-D4** significantly decreased the aggregate/monomer ratio to 0.387 ± 0.15 and 0.569 ± 0.08 (Figure 11), while **MB-D1** decreased the ratio to 0.7036 ± 0.07 vs. 1, although without reaching statistical significance. In the MCF-7 cell line, **MB-D2** decreased the aggregate/monomer ratio to 0.454 ± 0.02 vs. 1 (control), while **MB-D4** significantly decreased the ratio to 0.694 ± 0.14 (Figure 11). These results demonstrate the ability of compounds **MB-D2** and **MB-D4** to induce a selective decrease of Δψm in all tested cancer cell lines, revealing their potential to activate the intrinsic apoptotic pathway. In a similar manner, thiophene derivative F8, synthesized by the group of Swain et al. [18], induced caspase 3/7 activation and mitochondrial membrane depolarization and initiated the intrinsic apoptotic pathway as its mechanism of action in lymphoma (CCRF-CEM), leukemia (JURKAT and HL-60), breast adenocarcinoma (MDA-MB-231), pancreatic carcinoma (PANC-1), and malignant melanoma (A375) cell lines.

#### 2.2.5. Effects of **MB-D1**, **MB-D2**, and **MB-D4** Derivatives on ROS Production

**Figure 12:**
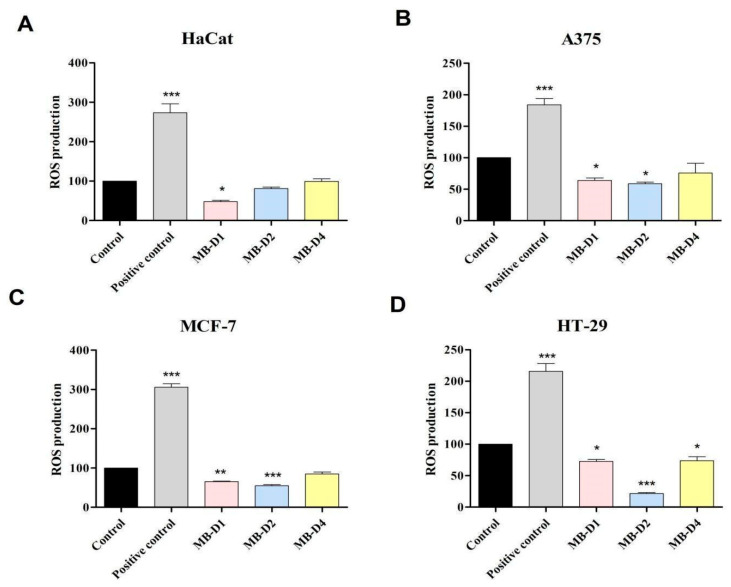
The effect of 48 h treatment with **MB-D1**, **MB-D2**, and **MB-D4** (IC_50_/100 μM) on ROS production in HaCaT (**A**), A375 (**B**), HT-29 (**C**), and MCF-7 (**D**) cell lines. The results are expressed as percentages in comparison with the control group (considered to be 100%). Data represent the mean  ±  SD; n = 3 per group. * *p* < 0.05, ** *p* < 0.01, and *** *p* < 0.001.

ROS are active byproducts of numerous cellular processes. In cancer cells, ROS are viewed as a “double-edged sword” due to their ability to promote cell proliferation, migration, angiogenesis, and drug resistance at low levels, while, at high levels, ROS are able to induce damage to nucleic acids, proteins, lipids, membranes, and organelles, thereby leading to cell death [27]. To investigate if the observed effects of our compounds are a result of modulating ROS levels in cancer cells, the ROS production in HaCaT, A375, HT-29, and MCF-7 cell lines after 48 h treatment with **MB-D1**, **MB-D2**, and **MB-D4** (IC_50_/100 μM) was measured using a 2′,7′–dichlorofluorescein diacetate (DCFDA) assay. Tert-Butyl Hydroperoxide (TBHP) was used as a positive control (Figure 12). The results show that in HaCaT cell lines, only **MB-D1** decreased ROS production (47.77 ± 5.88 vs. 100%), while in A375 and HT-29, **MB-D1** and **MB-D2**, respectively, decreased ROS production in a statistically significant manner as follows: 64.12 ± 6.548 and 58.89 ± 4.185 vs. 100% in the A375 cell line and 70.66 ± 5.722 and 21.52 ± 3.033 vs. 100% in the HT-29 cell line (Figure 12A–C). In MCF-7 cells, all the compounds decreased ROS production vs. the control (100%) as follows: 65.23 ± 2.243, 54.86 ± 5.070, and 85.22 ± 8.527 (Figure 12D).

The obtained results clearly show that compound **MB-D2** leads to the most significant decrease in ROS production in all tested cancer cell lines. Even though cancer cells may rely on increased ROS generation to regulate their phenotype, the scientific literature demonstrates that a further increase in ROS production severely affects their redox homeostasis and ultimately leads to cell death [28]. Thus, in the context of cancer cells, one would expect that an effective compound would have to significantly increase ROS production in order to have a strong cytotoxic effect. However, the decrease in ROS observed in our study may be attributed to the mitochondrial dysfunction and loss of Δψm that occurs during apoptosis. As suggested by various studies, this ROS reduction may reflect the downstream effects of mitochondrial depolarization rather than the direct inhibition of the ROS-mediated signaling, which can induce the activation of the apoptotic pathway [29,30,31,32].

## 3. Materials and Methods

### 3.1. Chemistry

Thionyl chloride (Acros Organics, Geel, Belgium), 5-bromothiophene-2-carboxylic acid (97%, AmBeed, Arlington Heights, IL, USA), 4-dimethylaminopyridine (99%, Merck, Damstadt, Germany); 2-aminothiazole (98%), 4-amino-1,5-dimethyl-2-phenyl-1,2 -dihydro-3H-pyrazol-3-one (98%), triethylamine, morpholine, dicyclohexylcarbodiimide, anhydrous dichloromethane—all from Sigma-Aldrich (St. Louis, MO, USA), 4-((methoxycarbonyl)phenyl)boronic acid (Bidepharm, Shanghai, China, 99.93%), anhydrous sodium sulphate (ChimReactiv, Bucuresti, Romania), Celite 545 (Carl Roth, Karlsruhe, Germany). All other chemicals or solvents were of analytical grade and were used as received.

The purity of products was determined by a combination of techniques, including thin-layer chromatography (TLC) on silica gel-coated aluminum plates and elemental analysis using Elementar UNICUBE. The compounds were purified by a gravitational column chromatograph packed with silica gel (400–600 mesh) from Merck (Darmstad, Germany) as the stationary phase.

NMR spectra were recorded on a Bruker Avance III 500 spectrometer (500 MHz for **^1^**H, 125 MHz for **^13^**C) at 298 °K, using CDCl_3_ or TDF-d8 as solvents and TMS as an internal standard. Chemical shifts are reported as ppm.

High-resolution mass spectral (HRMS) data were obtained on an Orbitrap IQ-X Mass Spectrometer from Thermo Scientific (San José, CA, USA) using electrospray ionization (ESI). The instrument was operated in positive-ion mode, and ion transfer parameters were optimized to enable detection of ions in a *m*/*z* range between 150 and 1000. The employed parameters are listed in Table S1. The system was externally calibrated using Pierce FlexMix Calibration Solution from Thermo Scientific. The data was collected positive MS scan or SIM (Single-Ion Monitoring) mode, with scanning and processed performed using Thermo Scientific Xcalibur 4.7 software.

#### 3.1.1. Synthesis of **MB-D1**

5-bromothiophene-2-carboxylic acid (2.00 g, 9.65 mmol) was dissolved in anhydrous DCM (30 mL) under an atmosphere of argon. When a clear solution was observed, 4-dimethylaminopyridine (0.12 g, 0.95 mmol) was added in one portion, and it was allowed to stir for 30 min. Then, a solution of dicyclohexylcarbodiimide (2.78 g, 13.50 mmol) in anhydrous DCM (20 mL) was added at 0 °C in a small portion, followed by the addition of one portion of 2-aminothiazole (1.16 g, 11.58 mmol). After 12 h at r.t., the reaction was quenched with distilled water (100 mL). The compound was extracted with DCM (3 × 100 mL). The collected organic layers were washed with water (2 × 100 mL), dried over Na_2_SO_4_, filtered, and concentrated. The crude product was purified by column chromatography (EtOAc:Hexane = 8:2, Rf = 0.72). Product **MB-D1** was obtained as a yellow solid (0.97 g, η = 35%).

**^1^H-NMR** (500 MHz, THF-*d*_8_) δ (ppm): 11.61 (s, 1H), 7.68 (d, *J* = 4.1 Hz, 1H), 7.30 (d, *J* = 3.6 Hz, 1H), 7.11 (d, *J* = 4.1 Hz, 1H), 6.95 (d, *J* = 3.6 Hz, 1H). The intense signals at 3.48 and 1.38 are from deuterated tetrahydrofuran, and the signal at 3.16 is from water. (Figure S9). 

**^13^C-NMR** (125 MHz, THF-*d*_8_) δ (ppm): 160.2, 141.5, 138.2, 132.8, 131.5, 120.8, 117.5, 114.6. (Figure S10)

**ESI-HR-MS**: *m*/*z* calc. for C_8_H_6_^79^BrN_2_OS_2_^+^, ([M]+H^+^): 288.90994, found: 288.91010; *m*/*z* calc. for C_8_H_6_^81^BrN_2_OS_2_^+^, ([M]+H^+^): 290.90790, found: 290.90787. (Figures S1 and S2). 

#### 3.1.2. Synthesis of **MB-D2**

5-bromothiophene-2-carboxylic acid (1.20 g, 5.80 mmol) was dissolved in DCM (10 mL), followed by the dropwise addition of thionyl chloride (SOCl_2_; 1.0 g, 610 μL, 8.4 mmol) within 30 min. The mixture was refluxed for 4 h. The solvent and the residual SOCl_2_ were removed by rotary evaporation to yield a yellow, solid mass. The product was washed with DCM (2 × 5 mL) to remove the trace of SOCl_2_ and was used immediately in the next step without further purification to prevent hydrolysis. 5-Bromothiophene-2-carbonyl chloride was dissolved in DCM (5 mL) and added dropwise to a mixture of DCM (10 mL), 4-amino-1,5-dimethyl-2-phenyl-1,2-dihydro-3H-pyrazol-3-one (0.45 g, 2.22 mmol), and TEA (0.45 g, 617 µL, 4.44 mmol) at 0 °C and under strong stirring. The reaction mixture was allowed to warm up to r.t. and stirred for another 48 h. Distilled water (50 mL) was added to quench the reaction, and saturated NaHCO_3_ (10 mL) was added to separate the phases. The product was extracted with DCM (3 × 10 mL). The combined organic layers were washed successively with saturated Na_2_CO_3_ (10 mL), distilled water (10 mL), and saturated NaCl (10 mL). The organic phase was dried over Na_2_SO_4_, and the solvent was removed. The crude product was purified by column chromatography (EtOAc:hexane = 8:2, Rf = 0.61) to yield **MB-D2** as a yellow solid (0.50 g, η = 39%).

**^1^H-NMR** (500 MHz, CHCl_3_-d) δ (ppm): 7.61 (d, *J* = 4.1 Hz, 2H), 7.41 (m, 2H), 7.31 (d, *J* = 2.2 Hz, 2H), 7.29 (m, 1H), 6.96 (d, *J* = 4.1 Hz, 2 H), 3.18 (s, 3H), 2.18 (s, 3H). (Figure S11).

**^13^C-NMR** (125 MHz, CHCl_3_-d) δ (ppm): 164.3, 161.4, 153.2, 137.4, 135.2, 134.1, 131.0, 129.5, 127.8, 125.1, 122.2, 108.4, 35.2, 10.8 (Figure S12). The full assignment of carbon atoms was performed on the basis of 2D correlation spectra (Figures S13–S15) and the results are presented on Table S2.

**ESI-HR-MS**: *m*/*z* calc. for C_21_H_16_^79^Br_2_N_3_O_3_S_2_^+^, ([M]+H^+^): 579.89943, found: 579.89966.m/z calc. for C_21_H_16_^79^Br^81^BrN_3_O_3_S_2_^+^, ([M]+H^+^): 581.89739, found: 581.89740. *m*/*z* calc. for C_21_H_16_^81^Br_2_N_3_O_3_S_2_^+^, ([M]+H^+^): 583.89534, found: 583.89496, the compound isotopes shows peaks at *m*/*z* 579, 581, and 583, with relative abundances of 1:2:1. (Figures S3 and S4).

#### 3.1.3. Synthesis of **MB-D3**

5-bromothiophene-2-carboxylic acid (2.00 g, 9.65 mmol) was dissolved in anhydrous DCM (30 mL) under an argon atmosphere. When a clear solution was observed, 4-dimethylaminopyridine (0.12 g, 0.96 mmol) was added in one portion, and it was stirred for 30 min. A solution of dicyclohexylcarbodiimide (2.78 g, 13.5 mmol) in anhydrous DCM (20 mL) was added in small portions at 0 °C for 5 min, followed by the addition of one portion of morpholine (1.00 g, 0.99 mL, 11.58 mmol). The reaction was carried out at r.t. overnight. Distilled water (100 mL) was added, and the organic phase was extracted in DCM (3X10 mL). The combined organic layers were dried over Na_2_SO_4_, and the solvent was evaporated. The crude product was purified by column chromatography (EtOAc, Rf = 0.65), yielding **MB-D3** as a white solid (0.80 g, η = 29%)

**^1^H-NMR** (500 MHz, CHCl_3_-d) δ (ppm): 6.99 (d, J = 3.9 Hz, 1H), 6.94 (d, J = 3.9 Hz, 1H), 3.67 (m, 8H). (Figure S16).

**^13^C NMR** (125 MHz, CHCl_3_-*d*) δ 162.3, 138.2, 129.7, 129.4, 116.8, 66.8. (Figure S17)

**ESI-HR-MS**: *m*/*z* calc. for C_9_H_11_Br^79^NO_2_S^+^, ([M]+H^+^): 275.96884, found: 275.96890. *m*/*z* calc. for C_9_H_11_Br^81^NO_2_S^+^, ([M]+H^+^): 277.96679, found: 277.96671. (Figures S5 and S6).

#### 3.1.4. Synthesis of **MB-D4**

5-Bromo-N-(5-bromothiophene-2-carbonyl)-N-(1,5-dimethyl-3-oxo-2-phenyl-2,3-dihydro-1H-pyrazol-4-yl)thiophene-2-carboxamide (**MB-D2**) (0.40 g, 0.69 mmol) and (4-(methoxycarbonyl)phenyl)boronic acid (0.27 g, 1.52 mmol) were added to a 50 mL round-bottom flask containing 1,4-dioxane (10 mL) as a solvent. The mixture was allowed to stir at r.t. for a few minutes under nitrogen flow. A solution of K_2_CO_3_ (0.61 g, 4.44 mmol dissolved in 2 mL H_2_O) and Pd(PPh_3_)_4_ catalyst (0.26 g, 0.22 mmol) was added to the reaction mixture under nitrogen. The resulting mixture was heated up to 95 °C and allowed to react at this temperature overnight. The cold mixture was diluted with EtOAc and filtered through Celite to remove the catalyst. The filtrate was washed with distilled water and brine and dried over Na_2_SO_4_, and the solvents were removed. The crude product was purified by column chromatography (EtOAc:hexane = 8:2, Rf = 0.54) to yield **MB-D4** as a slightly yellow solid (0.06 g, η = 13%)

**^1^H NMR** (500 MHz, CHCl_3_-*d*) δ (pp): 7.97 (m, 4H), 7.84 (d, *J* = 4.0 Hz, 2H), 7.59 (d, *J* = 8.4 Hz, 4H), 7.41 (t, *J* = 7.6 Hz, 2H), 7.33 (d, *J* = 7.8 Hz, 2H), 7.28 (d, *J* = 7.4 Hz, 1H), 7.26 (d, *J* = 4.1 Hz, 2H), 3.86 (s, 6H), 3.17 (s, 3H), 2.21 (s, 3H). (Figure S18).

**^13^C NMR** (125 MHz, CHCl_3_-*d*) δ (ppm): 166.5, 165.3, 161.7, 153.6, 150.8, 137.3, 136.5, 135.6, 134.5, 130.4, 130.2, 129.4, 127.6, 126.1, 125.0, 124.7, 109.6, 52.3, 35.4, 10.9. (Figure S19). The full assignment of carbon atoms was performed on the basis of 2D correlation spectra (Figures S20–S22) and the results are presented on Table S3.

**ESI-HR-MS**: *m*/*z* calc. for C_37_H_30_N_3_O_7_S_2_^+^, ([M]+H^+^): 692.15197, found: 692.15204. (Figures S7 and S8).

### 3.2. Biology

#### 3.2.1. Cell Culture

Immortalized human keratinocytes (HaCat) acquired from CLS Cell Lines Service GmbH (Eppelheim, Germany) and human breast adenocarcinoma (MCF-7), human colorectal adenocarcinoma (HT-29), and human malignant melanoma (A375) cells purchased from the American Type Culture Collection (ATCC, Lomianki, Poland) were selected for this study. The cells were received as frozen items and were stored in liquid nitrogen until further usage. HaCat and A375 cells were cultured and propagated in Dulbecco’s Modified Eagle Medium (DMEM) with added glucose, 10% fetal bovine serum (FBS), and a 1% penicillin/streptomycin mixture (100 IU/mL). McCoy’s 5A Medium was used to culture HT-29 cells, while MCF-7 cells were propagated in Eagle’s Minimum Essential Medium (EMEM) supplemented with 10% FBS, 1% antibiotic mixture, and 0.01 mg/mL human recombinant insulin. The cells were maintained in a humidified incubator at 37 °C with 5% CO_2_. After reaching 85–90% confluence, the cells were stimulated with the tested compounds at five increasing concentrations (10, 25, 50, 75, and 100 μΜ) for 48 h. The cell number was determined in the presence of Trypan blue using an automated cell-counting device (Thermo Fisher Scientific, Inc., Waltham, MA, USA).

#### 3.2.2. Cell Viability Assessment

The Alamar Blue colorimetric test was applied to assess the cell viability percentage of HaCat, MCF-7, HT-29, and A375 cells post stimulation with increasing concentrations (10, 25, 50, 75, and 100 μΜ) of four tested compounds—namely, **MB-D1**, **MB-D2**, **MB-D3**, and **MB-D4**, with 5-fluorouracil as a positive control—at the same concentrations for 48 h. The cells (1 × 10^4^) were seeded onto 96-well plates and incubated at 37 °C with 5% CO_2_ until reaching 85–90% confluence. The old medium was removed using an aspiration station, then replaced with a fresh medium containing the five concentrations of each tested compound. The tested concentrations (10, 25, 50, 75, and 100 μΜ) were prepared from a stock solution of 20 mM, and the final concentration of DMSO did not exceed 0.1%. After 48 h, the cells were stained with Alamar blue 0.01% and incubated for another 3 h. The absorbance measurements were determined at two wavelengths—570 and 600 nm—using a xMark™ Microplate Spectrophotometer (Bio-Rad, Hercules, CA, USA). All experiments were performed in triplicate.

#### 3.2.3. Cell Morphology Assessment

HaCaT, A375, MCF-7, and HT-29 cells were seeded in 6-well plates, treated with the tested compounds (100 μM **MB-D1**, **MB-D2**, **MB-D3**, and **MB-D4**), and incubated for 48 h. The cell morphology was analyzed using brightfield microscopy (Figure 2, Figure 3, Figure 4 and Figure 5). The cell morphology was also assessed using fluorescence microscopy (Figure 6, Figure 7, Figure 8 and Figure 9) but only that of compounds **MB-D1**, **MB-D2**, and **MB-D4** at their IC_50_ or at 100 μM if the calculated IC_50_ value was >100 μM. The nuclei and cell cytoskeleton were stained using Hoechst 34580 stain (H21486, Thermo Fisher Scientific, Boston, MA, USA) and F-actin (AB112127, Abcam, Cambridge, MA, USA). The apoptotic process was visualized by immunostaining with caspase 3/7 (C10423, Thermo Fisher Scientific, Boston, MA, USA). The cells were washed 3 times with cold PBS, fixed with 4% paraformaldehyde, permeabilized with Triton X/PBS 2%, blocked with 30% FCS in 0.01% Triton, and washed 3 times with cold PBS. After the staining, the cells were incubated at 4 °C in the dark. The morphological assessment was analyzed using the EVOS™ M5000 Imaging System (Thermo Fisher Scientific, Boston, MA, USA).

#### 3.2.4. Caspase-3/7 Assay

Apoptosis analysis was performed using the CellEvent™ Caspase-3/7 Green Detection Reagent kit (C10423, Thermo Fisher Scientific, Inc., Waltham, MA, USA). The stained cells treated with **MB-D1**, **MB-D2**, and **MB-D4** (at their IC_50_/100 μM) were analyzed live using an automated fluorescent cell counter equipped with a GFP filter set (Countess™ 3 FL Automated Cell Counter, Thermo Fisher Scientific, Inc., Waltham, MA, USA), following the protocol described by the manufacturer (https://www.thermofisher.com/order/catalog/product/C10423 (accessed on 2 September 2024)). The detection method is based on the ability of the regent’s dye to bind to DNA and produce a fluorogenic response (absorption/emission: 502/530 nm). The binding of the dye is possible only when the linked four-amino-acid peptide (DEVD), which inhibits the dye’s ability to bind to DNA, is cleaved due to caspase-3 or caspase-7 activation.

#### 3.2.5. JC-1 Assay

The mitochondrial membrane potential was assessed using a JC1 Kit (JC1- Mitochondrial Membrane Potential Assay Kit ab113850, Abcam, Cambridge, MA, USA) according to the manufacturer’s specifications (https://www.abcam.com/en-ro/products/assay-kits/jc-1-mitochondrial-membrane-potential-assay-kit-ab113850 (accessed on 1 October 2024)). The stained cells treated with **MB-D1**, **MB-D2**, and **MB-D4** (at IC_50_/100 μM) were analyzed using a multimode microplate reader equipped to measure fluorescence intensity (Varioskan™ LUX multimode microplate reader, Thermo Fisher Scientific, Inc., Waltham, MA, USA). The cationic JC-1 dye exhibits a potential-dependent accumulation in mitochondria, as observed by the shift in fluorescence emission from green to red (Ex/Em: 535/590 nm).

#### 3.2.6. H_2_DCDFA Assay

The production of ROS in treated cells (IC_50_/100 μM of **MB-D1**, **MB-D2**, and **MB-D4**) was evaluated using a cell-permeant 2′,7′-dichlorodihydrofluorescein diacetate (H_2_DCFDA) kit (DCFDA-Cellular ROS Assay Kit/Reactive Oxygen Species Assay Kit, ab113851, Abcam, Cambridge, MA, USA). ROS production was quantified at Ex/Em: 485/535 nm using a multimode microplate reader equipped to measure fluorescence intensity (Varioskan™ LUX multimode microplate reader, Thermo Fisher Scientific, Inc., Waltham, MA, USA), following the protocol described by the manufacturer (DCFDA/H2DCFDA-Cellular ROS Assay Kit (ab113851)|Abcam, Cambridge, UK). The detection method is based on the ability of the nonfluorescent H2DCFDA to convert to highly fluorescent 2′,7′-dichlorofluorescein (DCF) when cleaved by ROS.

### 3.3. Statistical Analysis

The statistical analysis was carried out by performing one-way ANOVA, followed by Dunnett’s post-test (GraphPad Prism version 6.0.0, GraphPad Software, San Diego, CA, USA). The IC_50_ values were calculated using GraphPad Prism6 software (GraphPad Software, San Diego, CA, USA) based on the correlation between the log[concentration] and cell viability. The differences between the groups were considered significant if *p* < 0.05, as follows: * *p* < 0.05, ** *p* < 0.01, and *** *p* < 0.001.

## 4. Conclusions

This study demonstrates that novel synthesized compounds **MB-D1**, **MB-D2**, and **MB-D4** exhibit a significant cytotoxic effect on A375, HT-29, and MCF-7 cancer cell lines. These compounds also induced cellular morphological changes consistent with apoptotic cell death. As confirmed by subsequent tests that revealed the loss of the mitochondrial membrane potential (Δψm) and the activation of caspase-3/7, treatment with **MB-D2** and **MB-D4** seem to induce apoptosis through the intrinsic mitochondrial pathway. The compounds also decrease ROS levels, an effect that reflects the downstream effects of mitochondrial depolarization rather than the direct inhibition of the ROS-mediated signaling pathway normally involved in the activation of the apoptotic pathway. Moreover, of the three compounds, **MB-D2** proved to be the most cytotoxic and effective in terms of caspase 3/7 activation, mitochondrial depolarization, and decreasing ROS production; these effects did not occur in normal HaCaT cells, revealing that **MB-D2** has a high selectivity against cancer cells. Taken together, all these findings warrant further investigation in order to fully elucidate their underlying mechanisms of action and their therapeutic potential in cancer treatment. Although the newly synthesized thiophene derivatives demonstrated moderate cytotoxicity in the micromolar range, these findings serve as a valuable starting point for further structural optimization. Future work will focus on improving potency and identifying specific pharmacological targets to guide rational design and enhance the biological relevance of these compounds.

## Figures and Tables

**Table 1:** IC_50_ values of **MB-D1**, **MB-D2**, **MB-D3**, and **MB-D4** against healthy and cancer cell lines.

	MB-D1	MB-D2	MB-D3	MB-D4
HaCaT	74.79	>100	>100	>100
A375	67.57	32.36	>100	84.3
MCF-7	>100	93.33	>100	>100
HT-29	>100	65.63	>100	>100

The compounds displaying IC_50_ values over 100 μΜ were considered ineffective.

## Data Availability

Data are contained within the article and Supplementary Materials.

## Supplementary Materials

The following supporting information can be downloaded at: https://www.mdpi.com/article/10.3390/ijms26146823/s1.

## References

[1] Shah R., Verma P. (2018). Therapeutic Importance of Synthetic Thiophene. Chem. Cent. J..

[2] Martins P., Jesus J., Santos S., Raposo L., Roma-Rodrigues C., Baptista P., Fernandes A. (2015). Heterocyclic Anticancer Compounds: Recent Advances and the Paradigm Shift towards the Use of Nanomedicine’s Tool Box. Molecules.

[3] Archna, Pathania S., Chawla P.A. (2020). Thiophene-Based Derivatives as Anticancer Agents: An Overview on Decade’s Work. Bioorg. Chem..

[4] Bray F., Laversanne M., Sung H., Ferlay J., Siegel R.L., Soerjomataram I., Jemal A. (2024). Global Cancer Statistics 2022: GLOBOCAN Estimates of Incidence and Mortality Worldwide for 36 Cancers in 185 Countries. CA. Cancer J. Clin..

[5] Chaib S., López-Domínguez J.A., Lalinde-Gutiérrez M., Prats N., Marin I., Boix O., García-Garijo A., Meyer K., Muñoz M.I., Aguilera M. (2024). The Efficacy of Chemotherapy Is Limited by Intratumoral Senescent Cells Expressing PD-L2. Nat. Cancer.

[6] Yahya E.B., Alqadhi A.M. (2021). Recent Trends in Cancer Therapy: A Review on the Current State of Gene Delivery. Life Sci..

[7] Thakur S., Kumar D., Jaiswal S., Goel K.K., Rawat P., Srivastava V., Dhiman S., Jadhav H.R., Dwivedi A.R. (2025). Medicinal Chemistry-Based Perspectives on Thiophene and Its Derivatives: Exploring Structural Insights to Discover Plausible Druggable Leads. RSC Med. Chem..

[8] Chaudhary A., Kumar S. (2012). Biological Diversity of Thiophene: A Review. J. Adv. Sci. Res..

[9] Yılmaz C., Othman Pirdawid A., Fidan Babat C., Konuş M., Çetin D., Kıvrak A., Algso M.A.S., Arslan Ş., Mutlu D., Otur Ç. (2022). A Thiophene Derivative, 2-Bromo-5-(2-(Methylthio)Phenyl)Thiophene, Has Effective Anticancer Potential with Other Biological Properties. ChemistrySelect.

[10] Bukhari S.N.A. (2022). Synthesis and Evaluation of New Chalcones and Oximes as Anticancer Agents. RSC Adv..

[11] Li T., Wang J., Feng L., Zhou Q., Xie Q., Shen Y., Ji R., Liu X., Wang Y., Hu C. (2024). Discovery of Novel Thiophene-3-Carboxamide Derivatives as Potential VEGFR-2 Inhibitors with Anti-Angiogenic Properties. Bioorg. Chem..

[12] Akatsuka A., Kojima N., Okamura M., Dan S., Yamori T. (2016). A Novel Thiophene-3-Carboxamide Analog of Annonaceous Acetogenin Exhibits Antitumor Activity via Inhibition of Mitochondrial Complex I. Pharmacol. Res. Perspect..

[13] Gulipalli K.C., Bodige S., Ravula P., Endoori S., Vanaja G.R., Suresh Babu G., Narendra Sharath Chandra J.N., Seelam N. (2017). Design, Synthesis, in Silico and in Vitro Evaluation of Thiophene Derivatives: A Potent Tyrosine Phosphatase 1B Inhibitor and Anticancer Activity. Bioorg. Med. Chem. Lett..

[14] Shams H.Z., Mohareb R.M., Helal M.H., Mahmoud A.E. (2010). Novel Synthesis and Antitumor Evaluation of Polyfunctionally Substituted Heterocyclic Compounds Derived from 2-Cyano-N-(3-Cyano-4,5,6,7-Tetrahydrobenzo[b]Thiophen-2-Yl)-Acetamide. Molecules.

[15] Morales-Tenorio M., Lasala F., Garcia-Rubia A., Aledavood E., Heung M., Olal C., Escudero-Pérez B., Alonso C., Martínez A., Muñoz-Fontela C. (2024). Discovery of Thiophene Derivatives as Potent, Orally Bioavailable, and Blood–Brain Barrier-Permeable Ebola Virus Entry Inhibitors. J. Med. Chem..

[16] Hwu J.R., Panja A., Jayakumar S., Tsay S.-C., Tan K.-T., Huang W.-C., Hu Y.-C., Leyssen P., Neyts J. (2020). Enterovirus Inhibition by Hinged Aromatic Compounds with Polynuclei. Molecules.

[17] Shah R., Verma P.K. (2019). Synthesis of Thiophene Derivatives and Their Anti-Microbial, Antioxidant, Anticorrosion and Anticancer Activity. BMC Chem..

[18] Swain R.M., Sanchez A., Gutierrez D.A., Varela-Ramirez A., Aguilera R.J. (2023). Thiophene Derivative Inflicts Cytotoxicity via an Intrinsic Apoptotic Pathway on Human Acute Lymphoblastic Leukemia Cells. PLoS ONE.

[19] Ziegler U., Groscurth P. (2004). Morphological Features of Cell Death. News Physiol. Sci. Int. J. Physiol. Prod. Jointly Int. Union Physiol. Sci. Am. Physiol. Soc..

[20] Park W., Wei S., Kim B.-S., Kim B., Bae S.-J., Chae Y.C., Ryu D., Ha K.-T. (2023). Diversity and Complexity of Cell Death: A Historical Review. Exp. Mol. Med..

[21] Jan R., Chaudhry G.-E.-S. (2019). Understanding Apoptosis and Apoptotic Pathways Targeted Cancer Therapeutics. Adv. Pharm. Bull..

[22] Voisine C., Craig E.A., Zufall N., von Ahsen O., Pfanner N., Voos W. (1999). The Protein Import Motor of Mitochondria: Unfolding and Trapping of Preproteins Are Distinct and Separable Functions of Matrix Hsp70. Cell.

[23] Matsuyama S., Reed J.C. (2000). Mitochondria-Dependent Apoptosis and Cellular PH Regulation. Cell Death Differ..

[24] Kroemer G., Reed J.C. (2000). Mitochondrial Control of Cell Death. Nat. Med..

[25] Ricci J.-E., Gottlieb R.A., Green D.R. (2003). Caspase-Mediated Loss of Mitochondrial Function and Generation of Reactive Oxygen Species during Apoptosis. J. Cell Biol..

[26] Zamzami N., Marchetti P., Castedo M., Zanin C., Vayssière J.L., Petit P.X., Kroemer G. (1995). Reduction in Mitochondrial Potential Constitutes an Early Irreversible Step of Programmed Lymphocyte Death in Vivo. J. Exp. Med..

[27] Nakamura H., Takada K. (2021). Reactive Oxygen Species in Cancer: Current Findings and Future Directions. Cancer Sci..

[28] Sabharwal S.S., Schumacker P.T. (2014). Mitochondrial ROS in Cancer: Initiators, Amplifiers or an Achilles’ Heel?. Nat. Rev. Cancer.

[29] Redza-Dutordoir M., Averill-Bates D.A. (2016). Activation of Apoptosis Signalling Pathways by Reactive Oxygen Species. Biochim. Biophys. Acta.

[30] Zorov D.B., Juhaszova M., Sollott S.J. (2014). Mitochondrial Reactive Oxygen Species (ROS) and ROS-Induced ROS Release. Physiol. Rev..

[31] Kowaltowski A.J., de Souza-Pinto N.C., Castilho R.F., Vercesi A.E. (2009). Mitochondria and Reactive Oxygen Species. Free Radic. Biol. Med..

[32] Li X., Fang P., Mai J., Choi E.T., Wang H., Yang X. (2013). Targeting Mitochondrial Reactive Oxygen Species as Novel Therapy for Inflammatory Diseases and Cancers. J. Hematol. Oncol..

