# Supplementary material for: Novel Compounds Featuring a Thiophene Carboxamide Scaffold: Synthesis, Characterization and Antiproliferative Evaluation
Source: Int J Mol Sci. 2025 Jul 16;26(14):6823. doi: 10.3390/ijms26146823 (PMC12295288; doi:10.3390/ijms26146823)
Supplement: Supplementary file 1 [file ijms-26-06823-s001.zip › ijms-3747454-supplementary material.pdf]

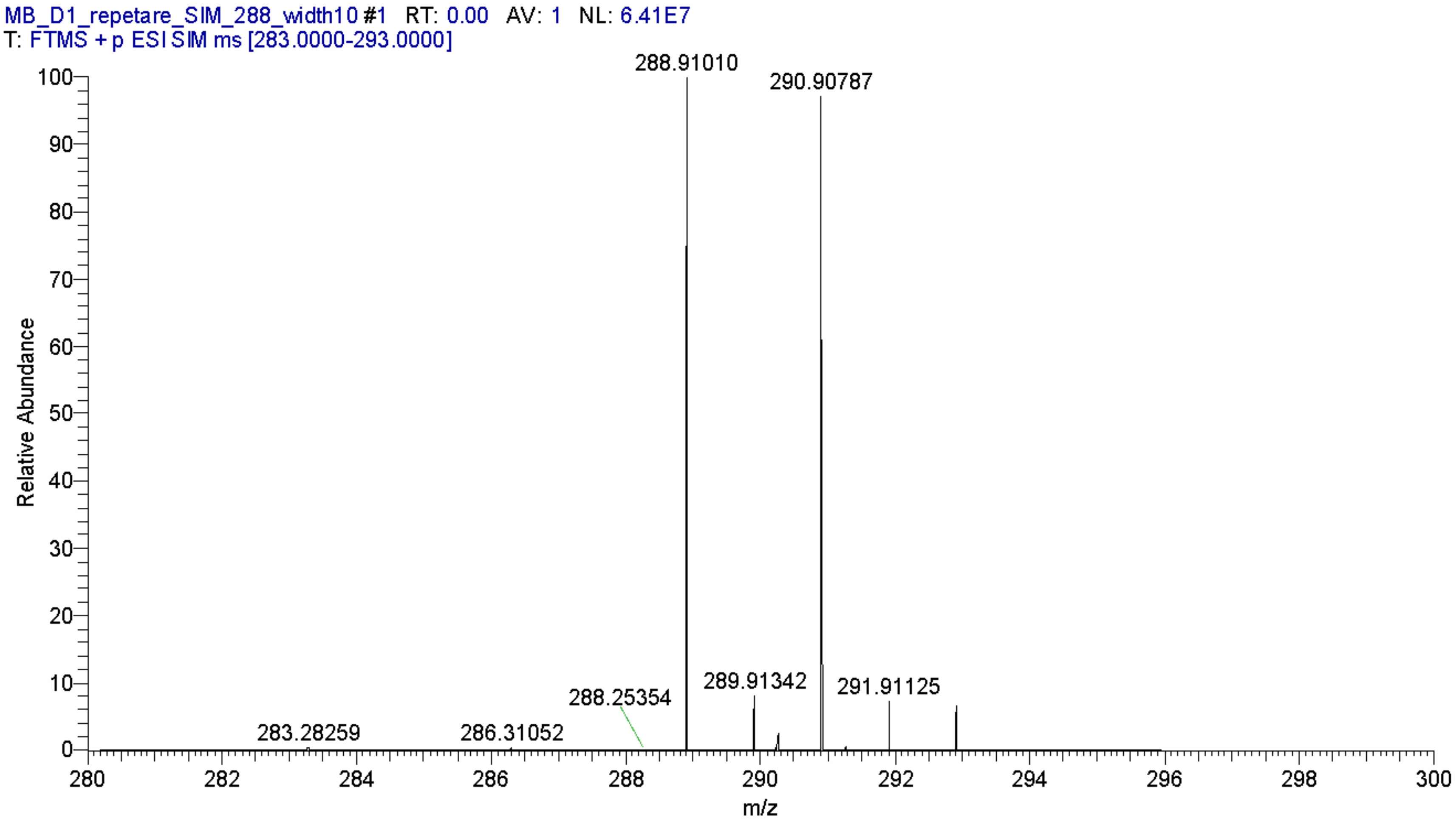


**Figure S1.** ESI-HR-MS spectrum of **MB-D1**

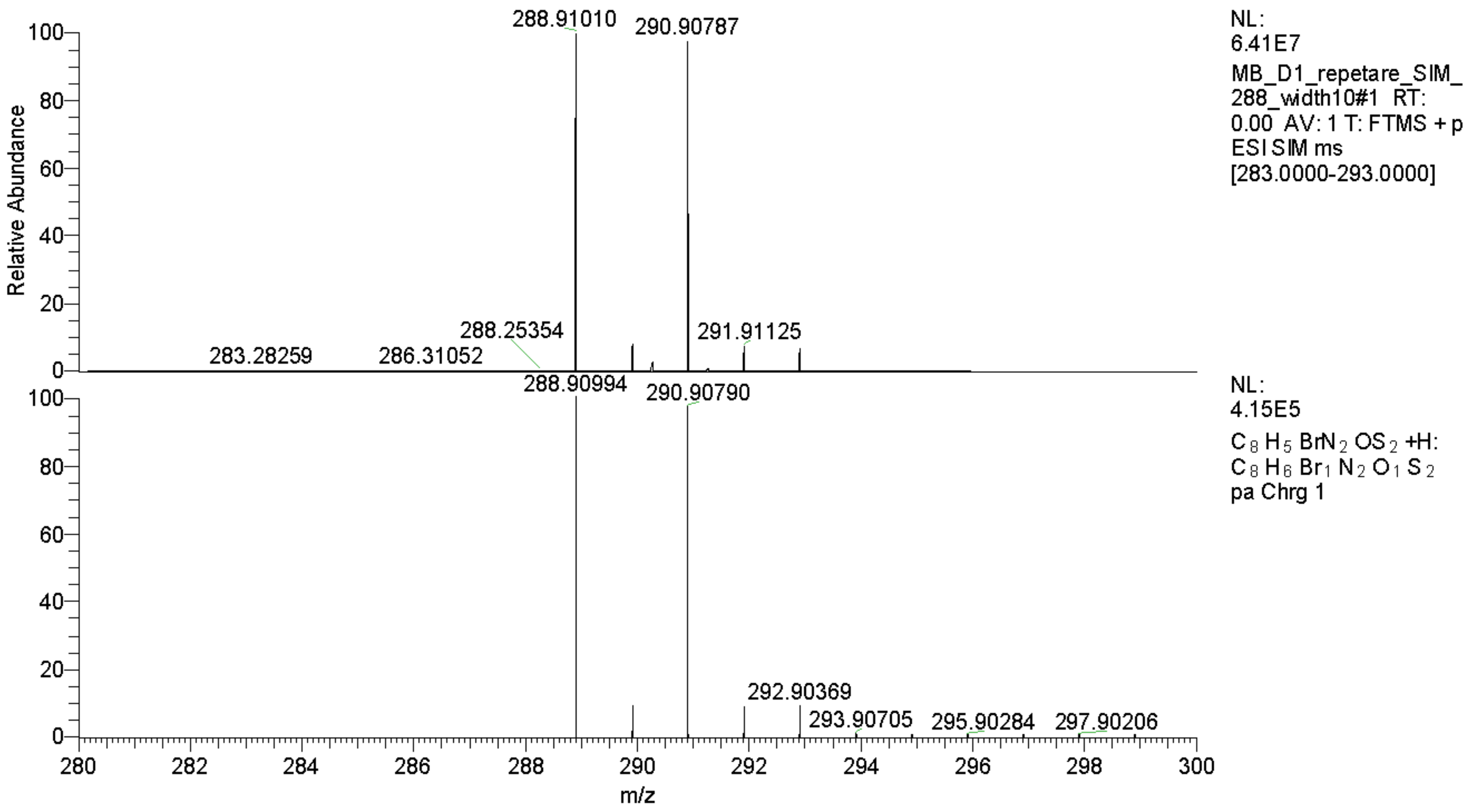


**Figure S2.** Mass analysis of **MB-D1**: ESI-HR-MS experimental spectrum (top); isotope simulation for predicted compound (bottom)

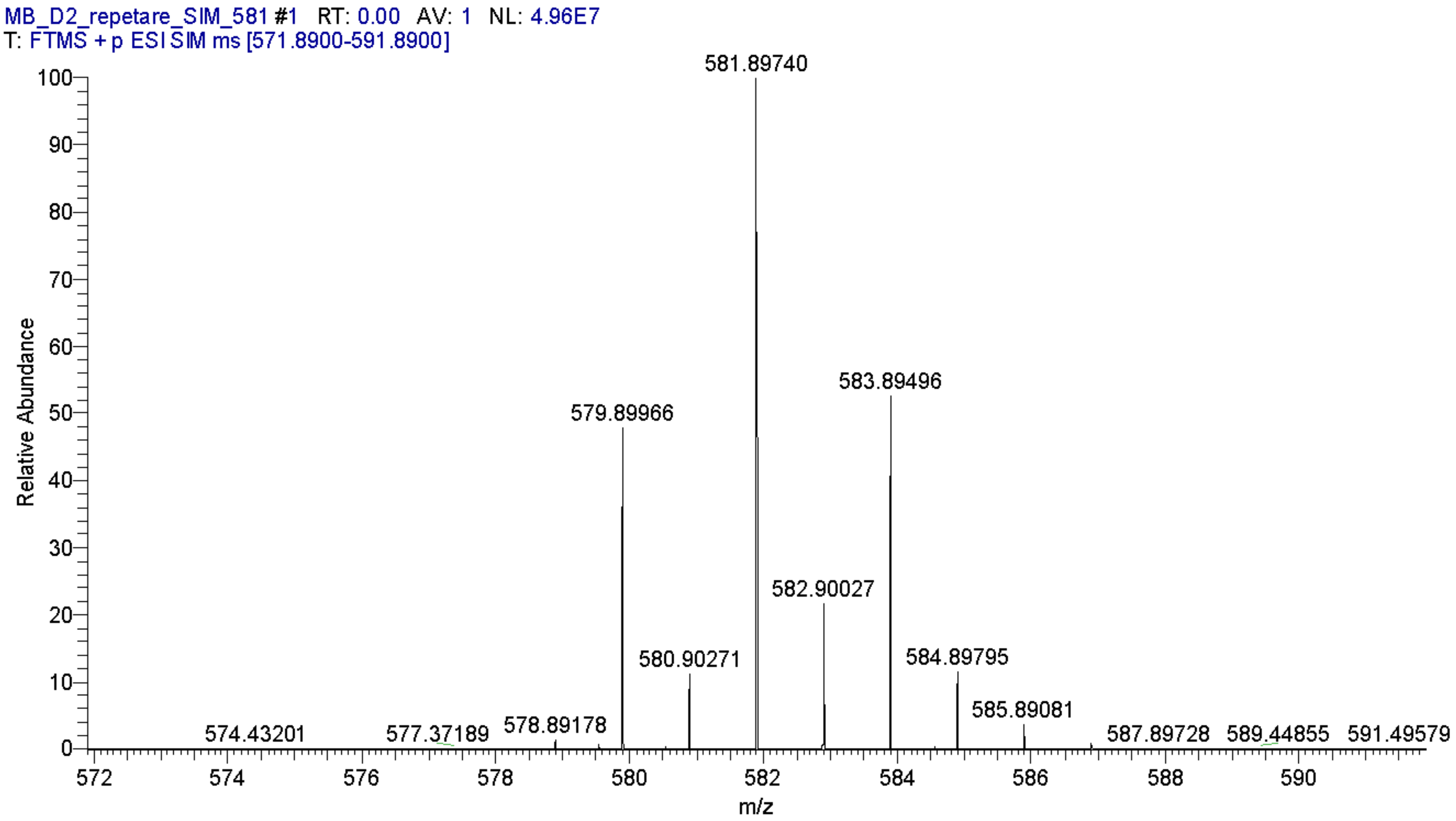


**Figure S3.** ESI-HR-MS spectrum of **MB-D2**

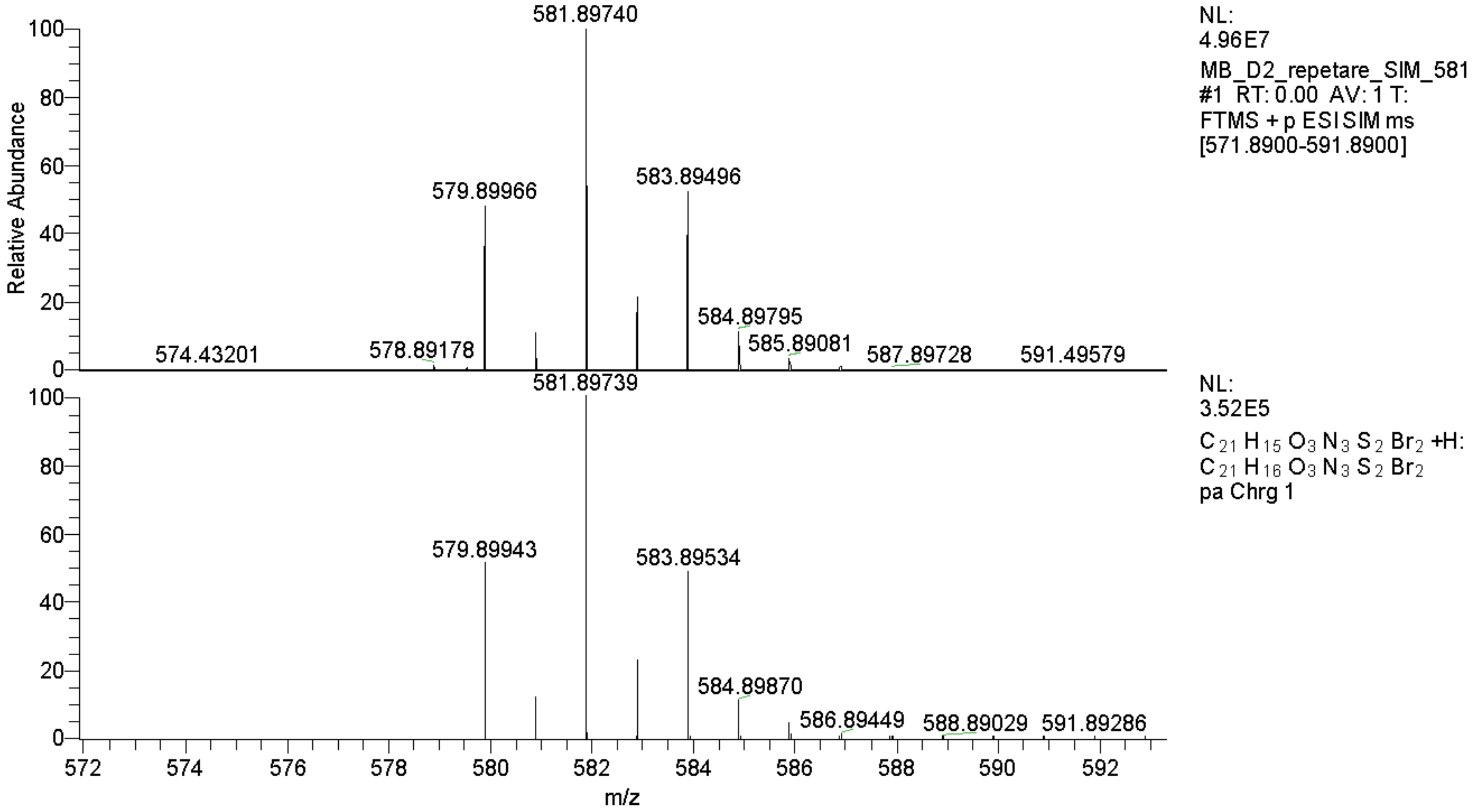


**Figure S4.** Mass analysis of **MB-D2**: ESI-HR-MS experimental spectrum (top); isotope simulation for predicted compound (bottom)

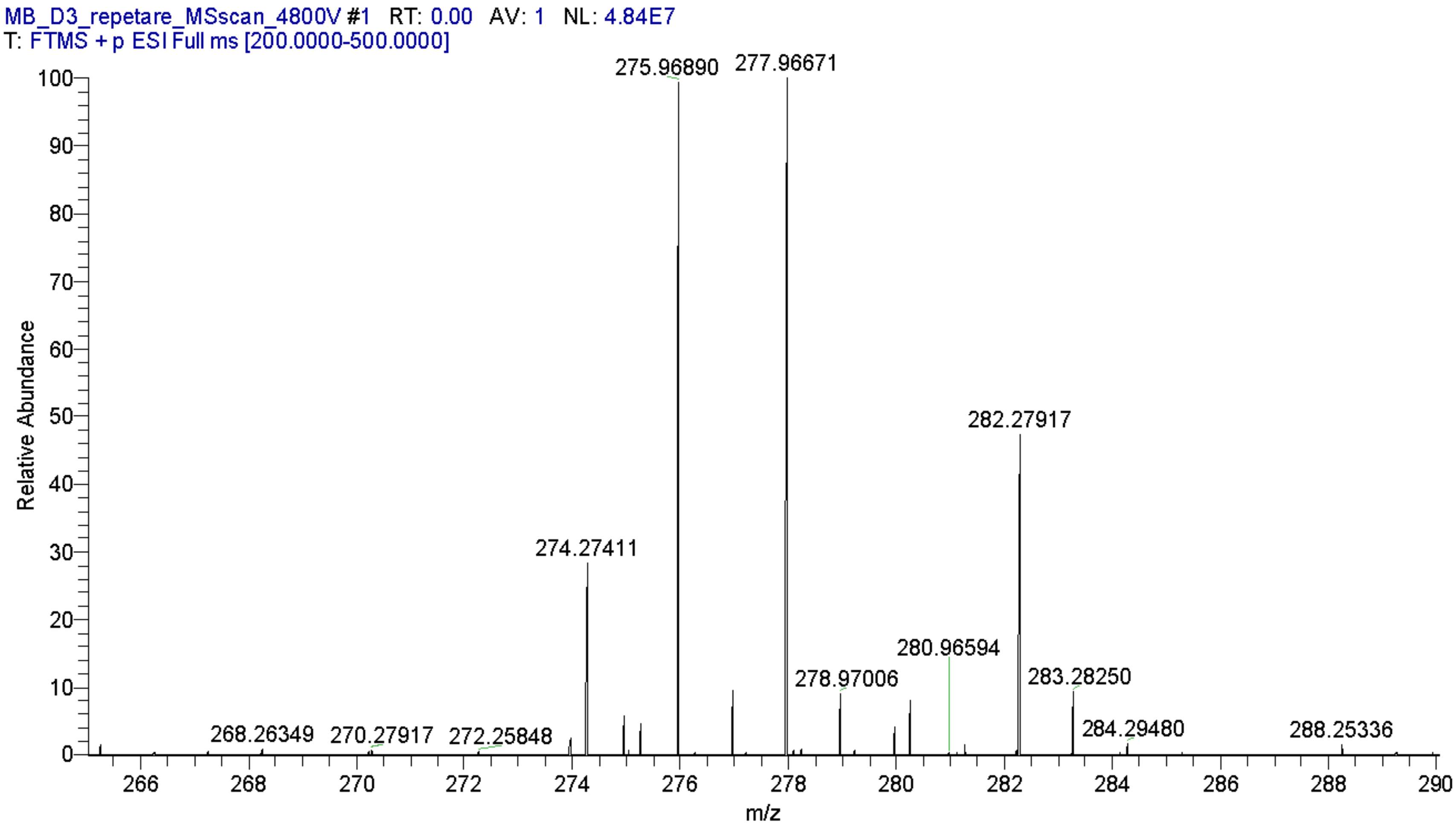


**Figure S5.** ESI-HR-MS spectrum of **MB-D3**

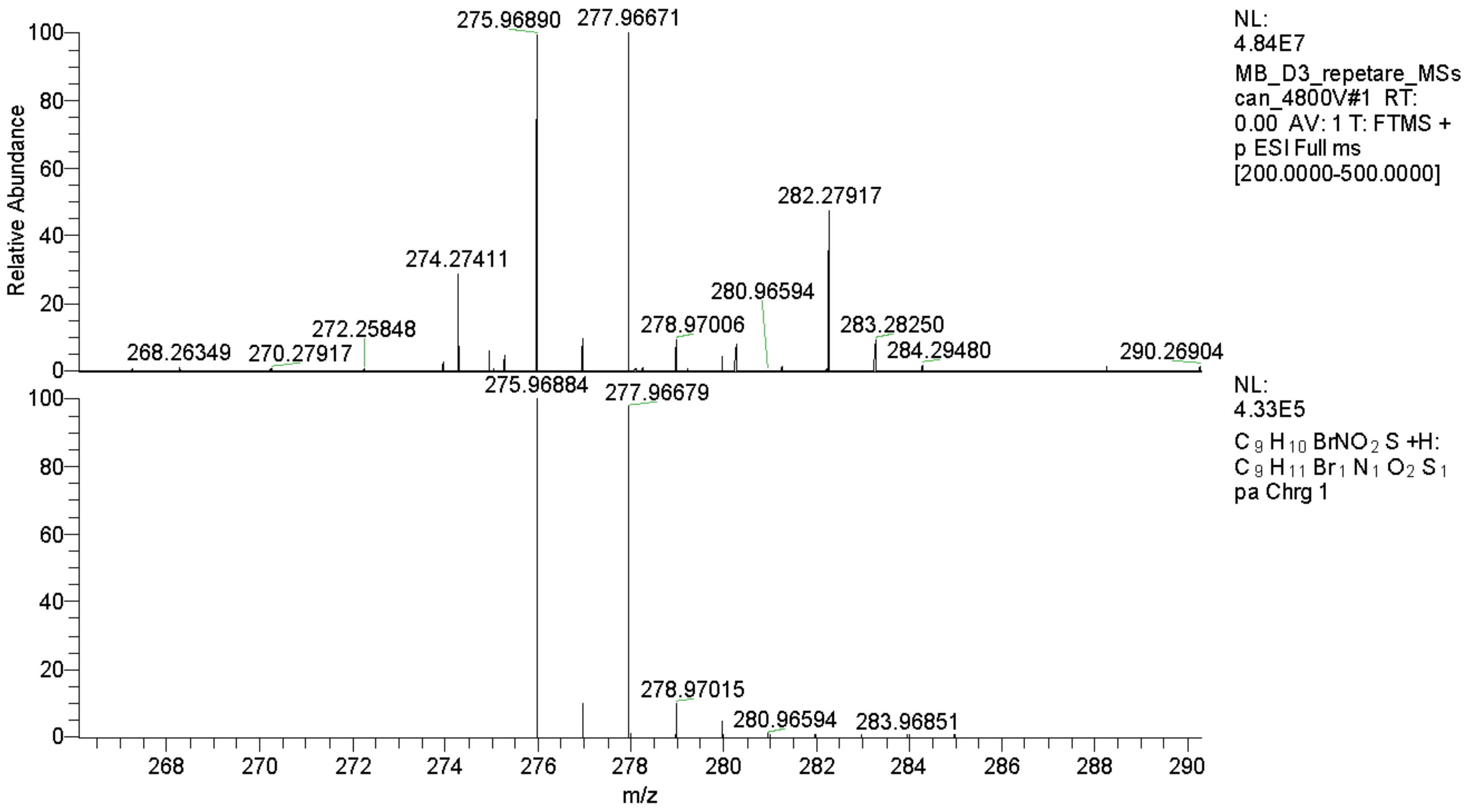


**Figure S6.** Mass analysis of **MB-D3**: ESI-HR-MS experimental spectrum (top); isotope simulation for predicted compound (bottom)

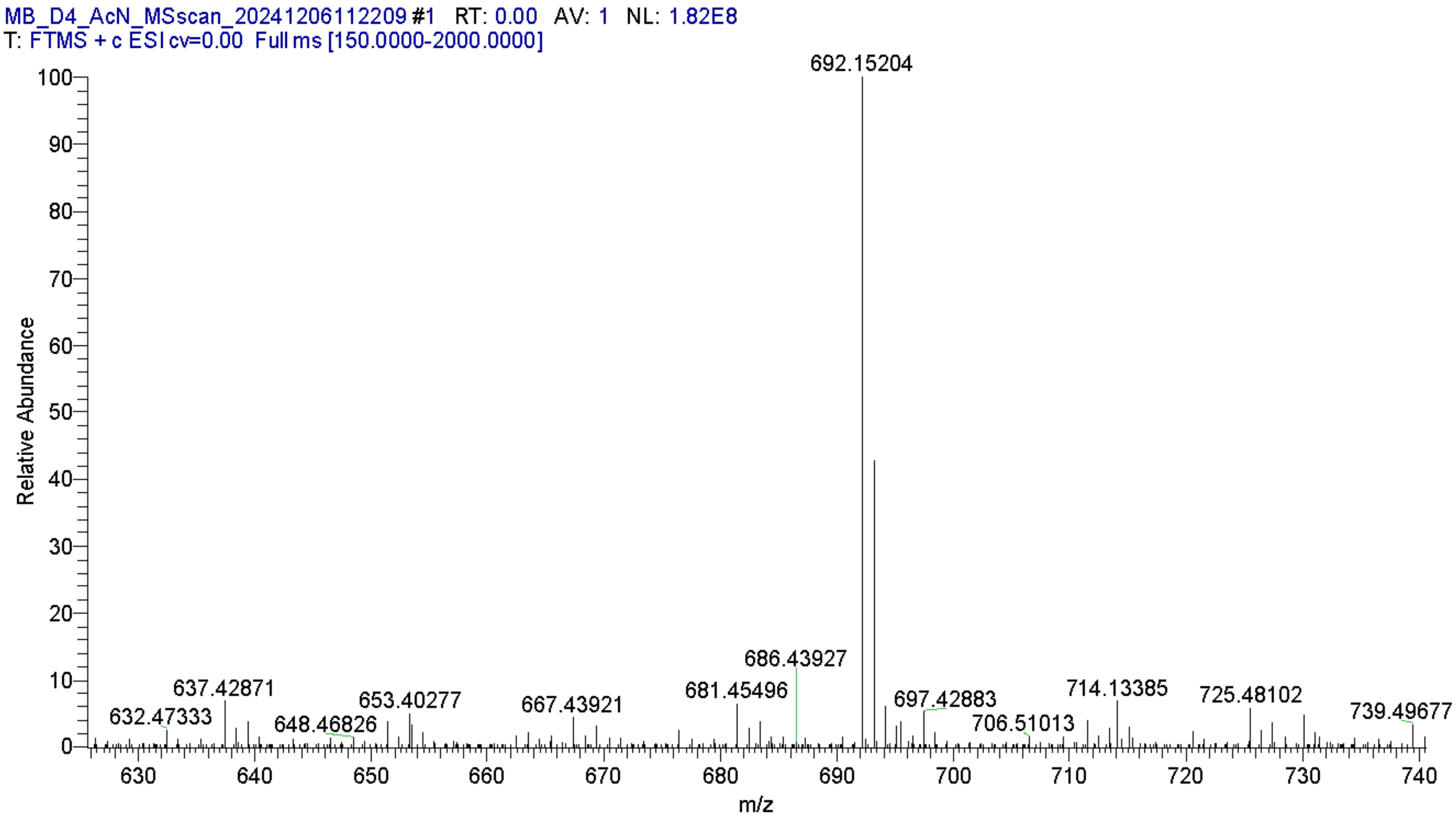


**Figure S7.** ESI-HR-MS spectrum of **MB-D4**

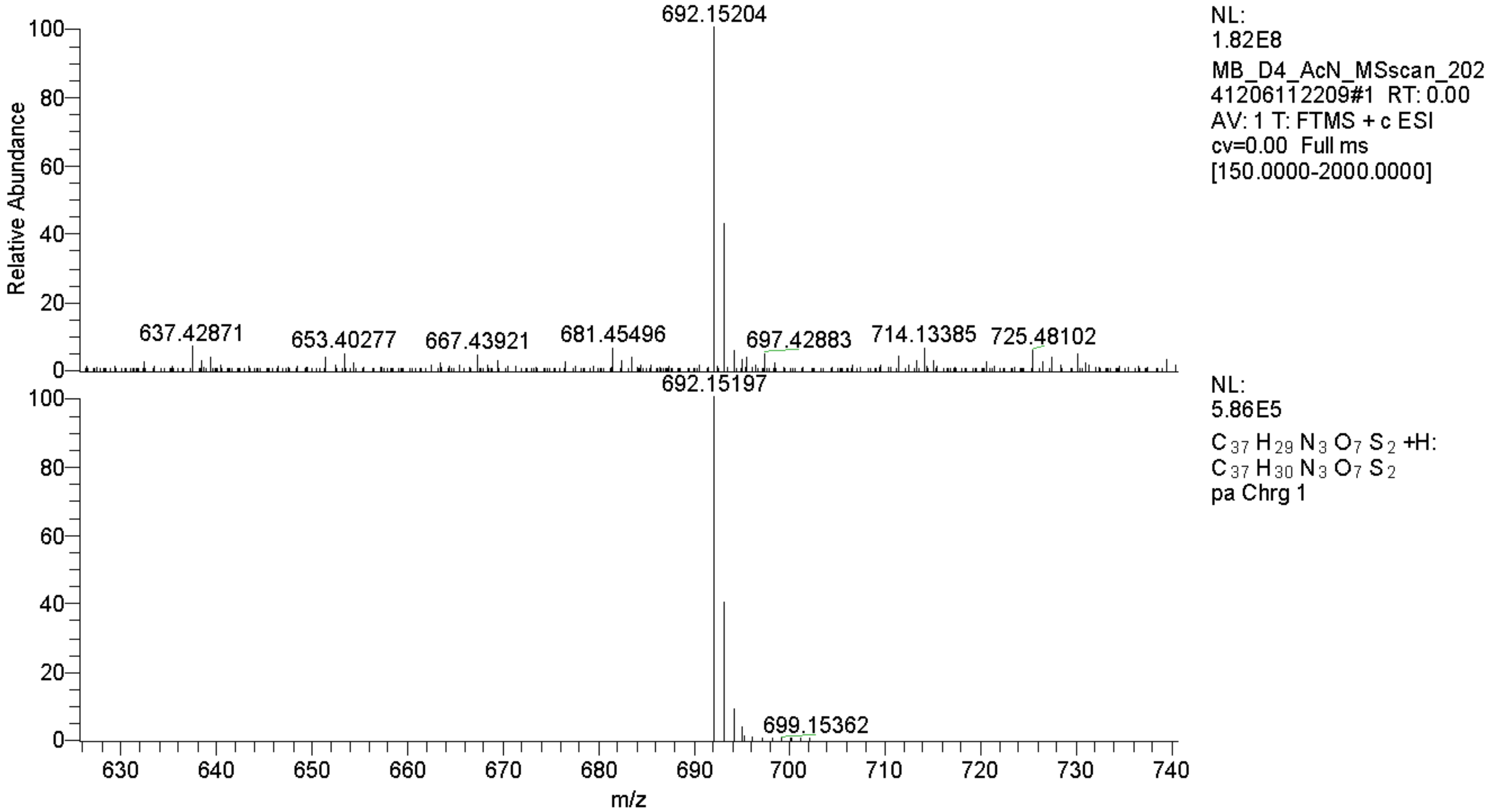


**Figure S8.** Mass analysis of **MB-D4**: ESI-HR-MS experimental spectrum (top); isotope simulation for predicted compound (bottom)

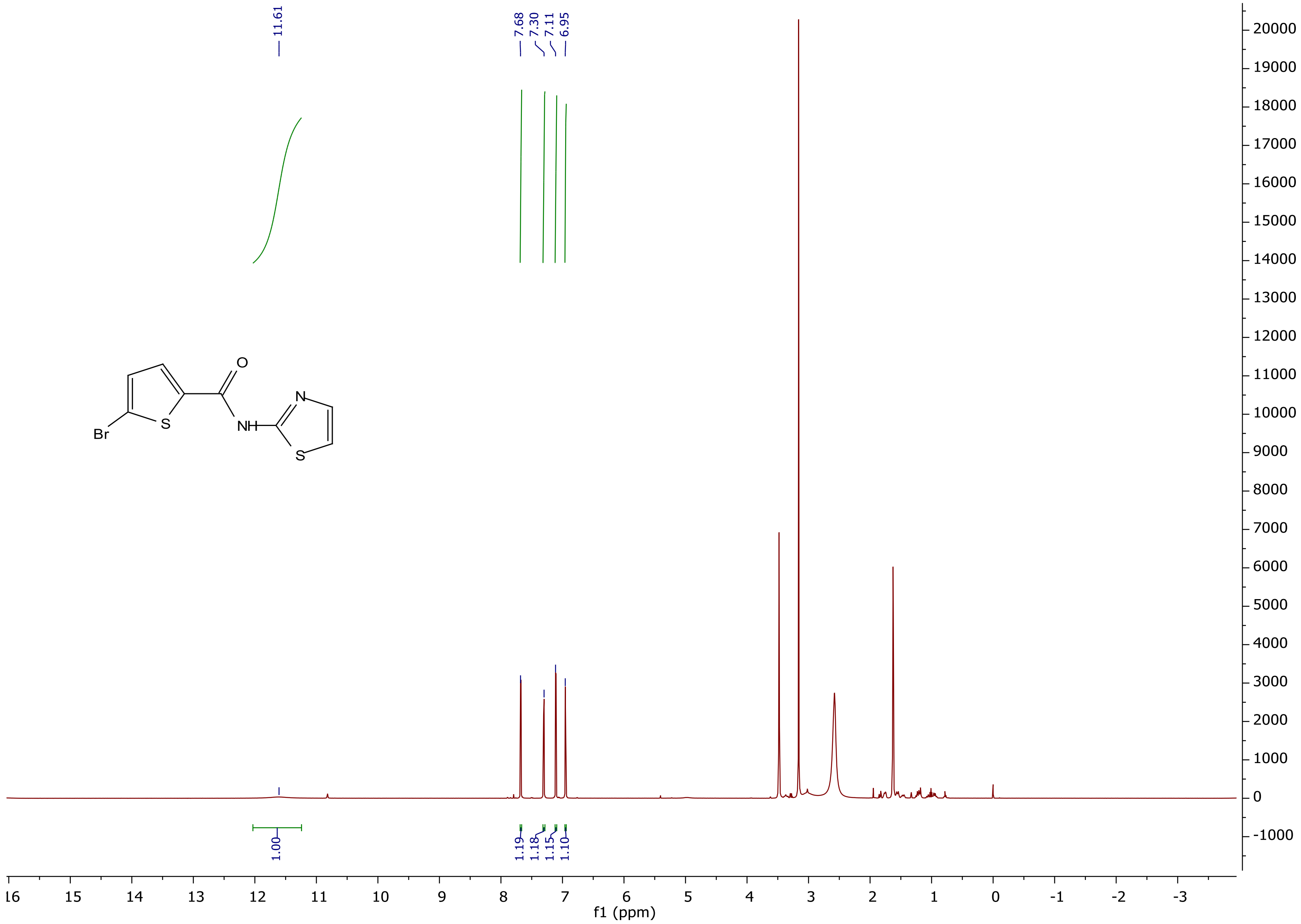


**Figure S9**. $^{1}$H-NMR spectrum of **MB-D1** in THF-*d8*, (500 MHz), 298K

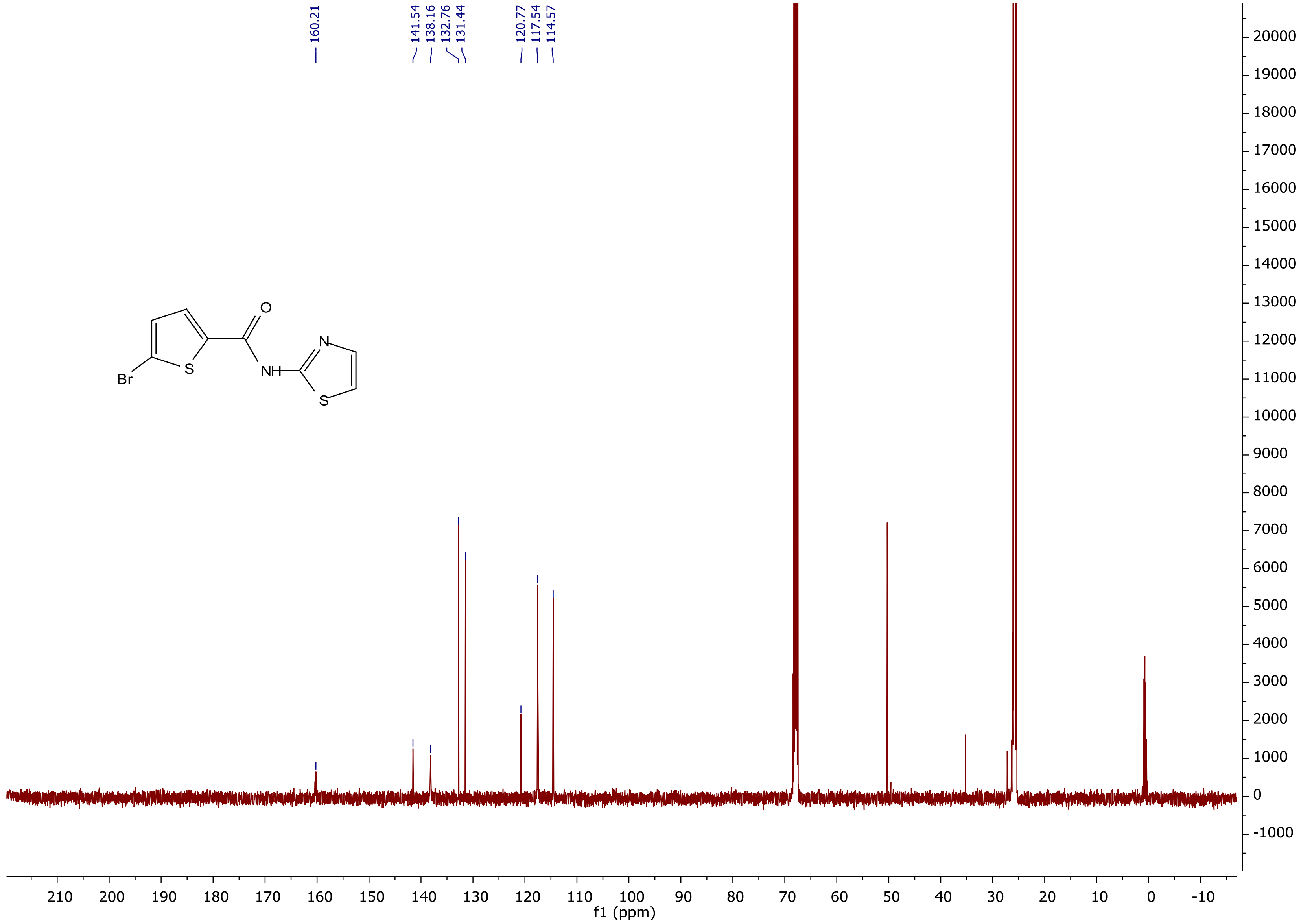


**Figure S10**. $^{13}$C-NMR spectrum of **MB-D1** in THF-*d8*, (125 MHz), 298K

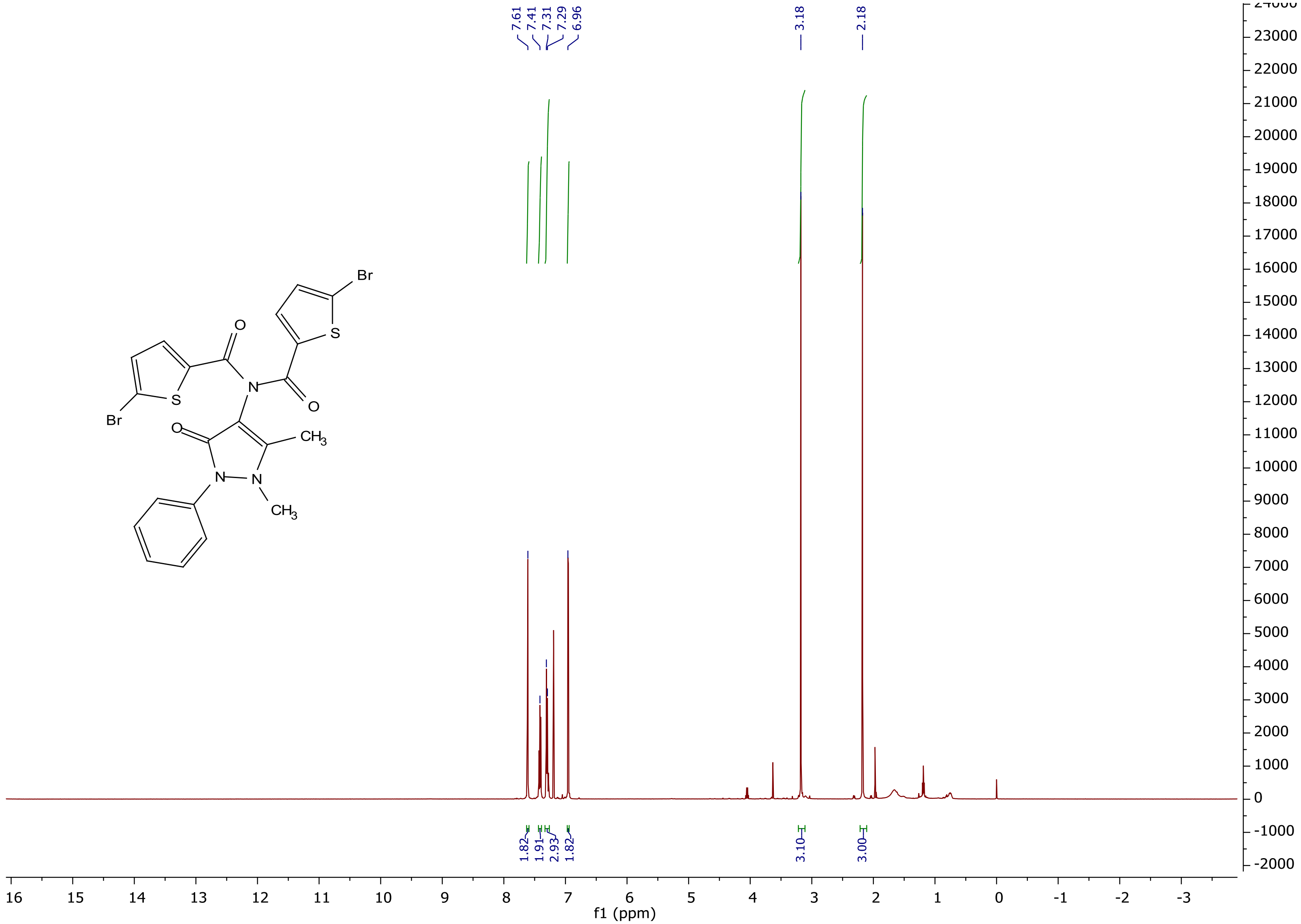

**Figure S11**. $^{1}$H-NMR spectrum of **MB-D2** in $CDCl_3$, (500 MHz), 298K

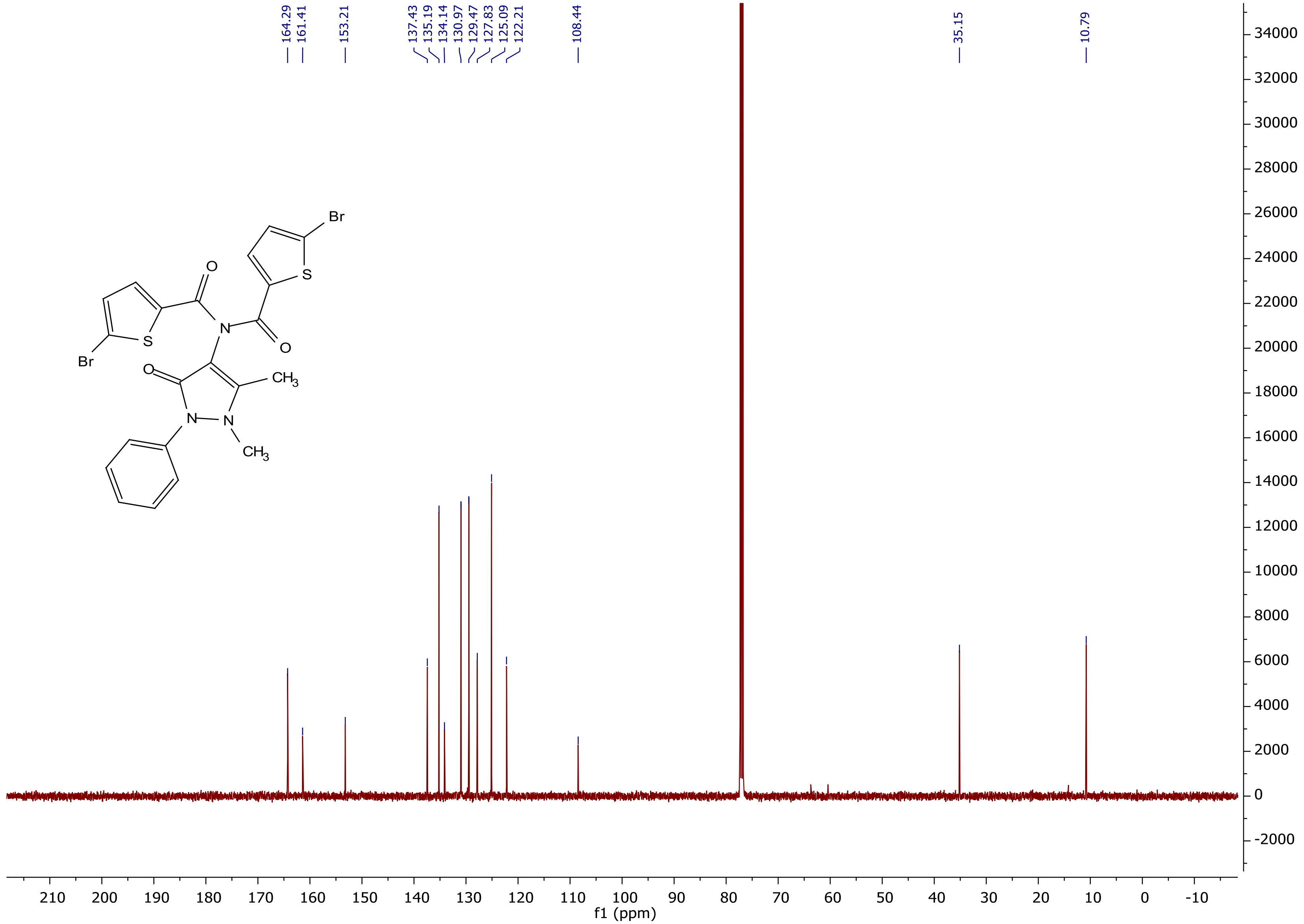


**Figure S12**. $^{13}$C-NMR spectrum of **MB-D2** in $CDCl_3$, (125 MHz), 298K

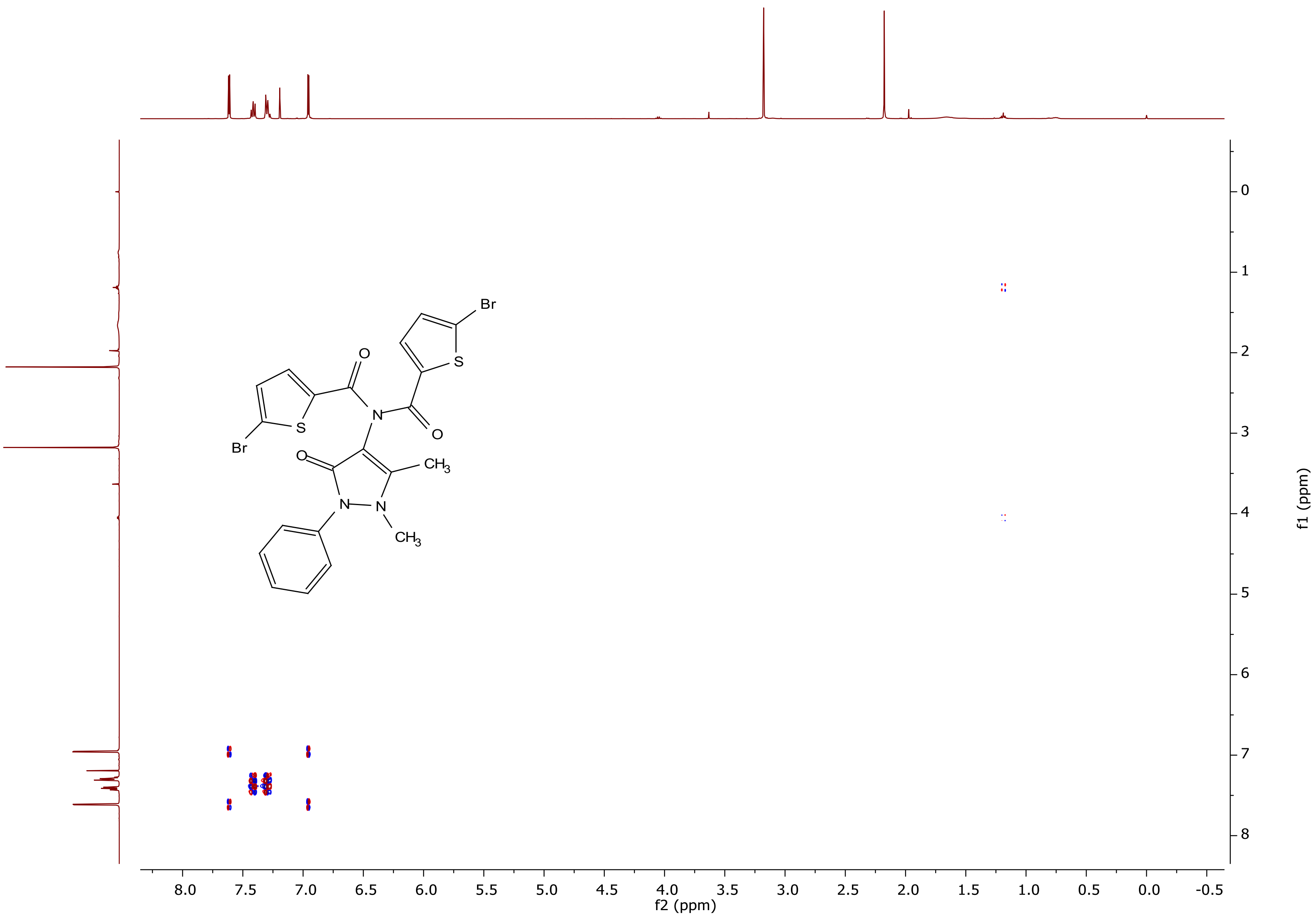


**Figure S13**. $^{1}$H-$^{1}$H-COSY of **MB-D2** in $CDCl_3$, (125 MHz), 298K

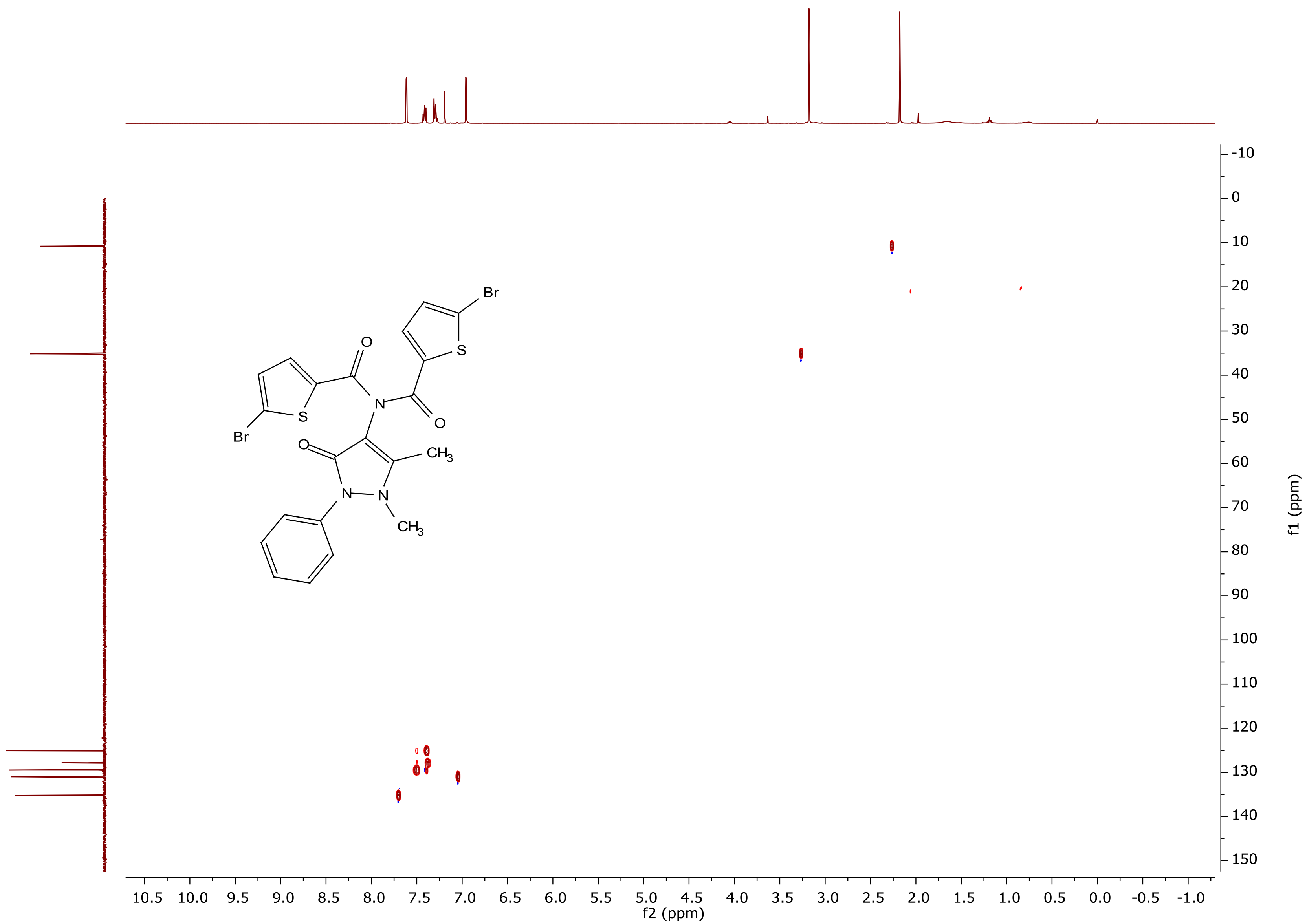


**Figure S14**. $^{1}H$-$^{13}C$-HSQC of **MB-D2** in $CDCl_3$, (125 MHz), 298K

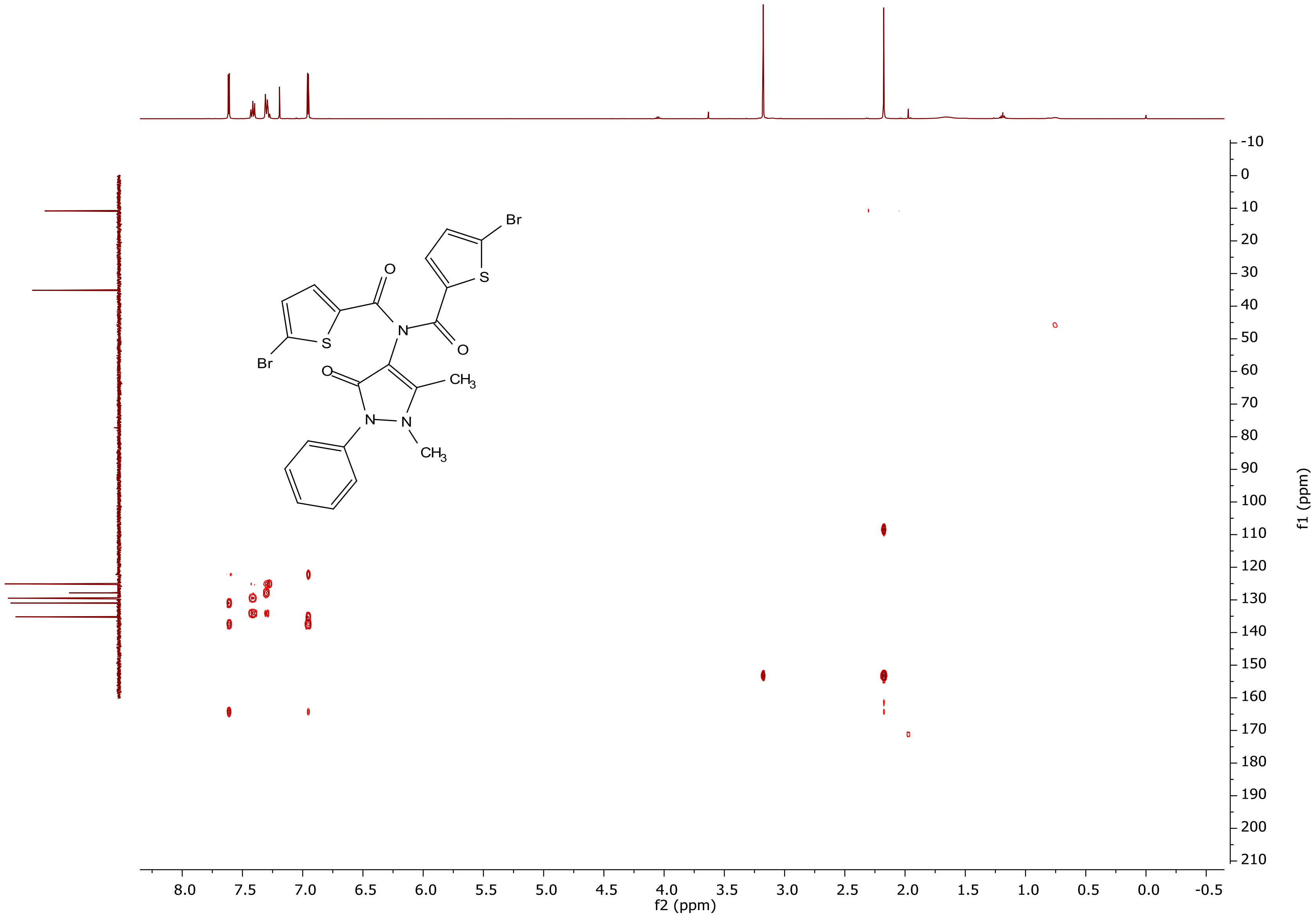


**Figure S15**. $^{1}H$-$^{13}C$-HMBC of **MB-D2** in $CDCl_3$, (125 MHz), 298K

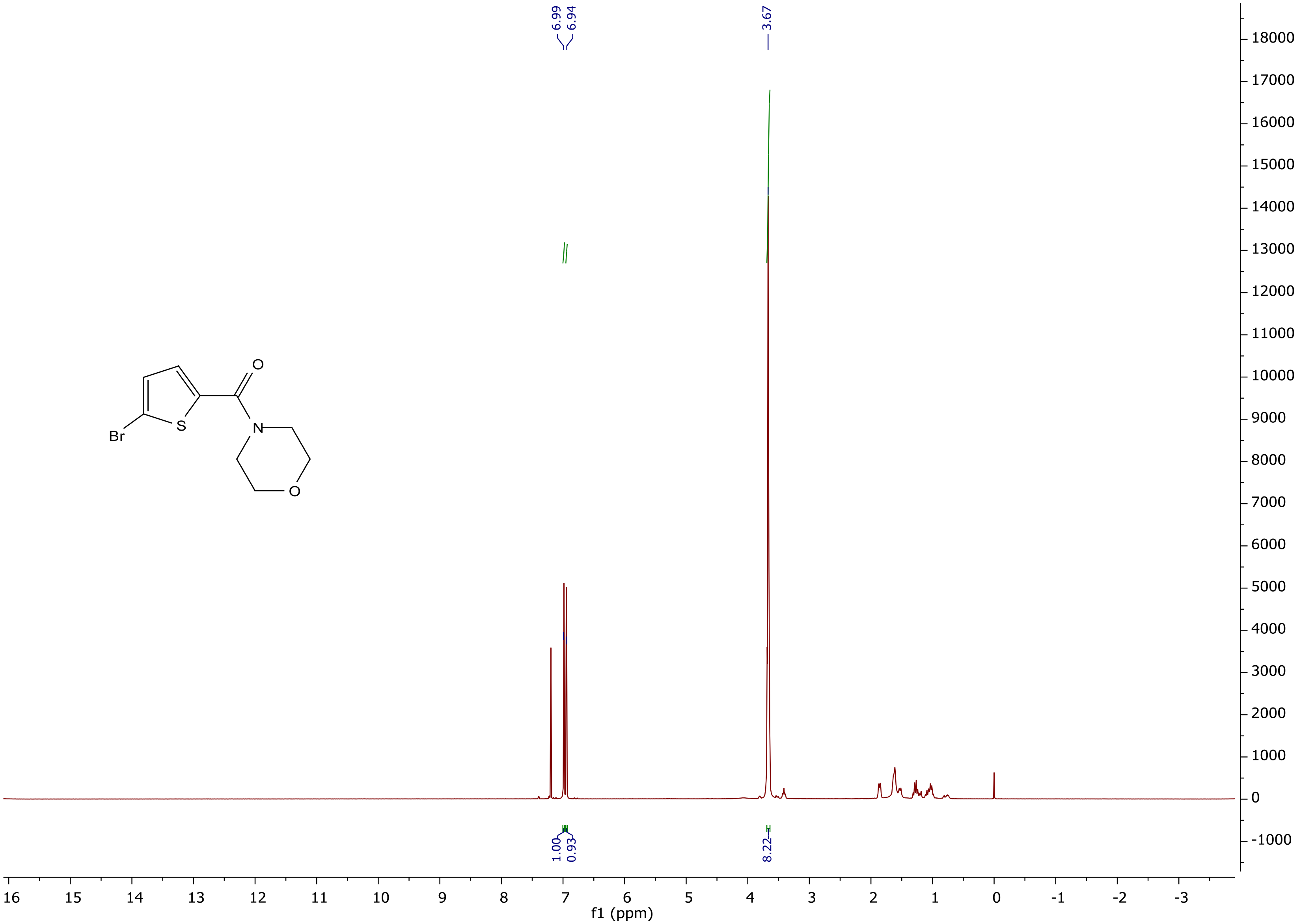


**Figure S16**. $^{1}$H-NMR spectrum of **MB-D3** in $CDCl_3$, (500 MHz), 298K

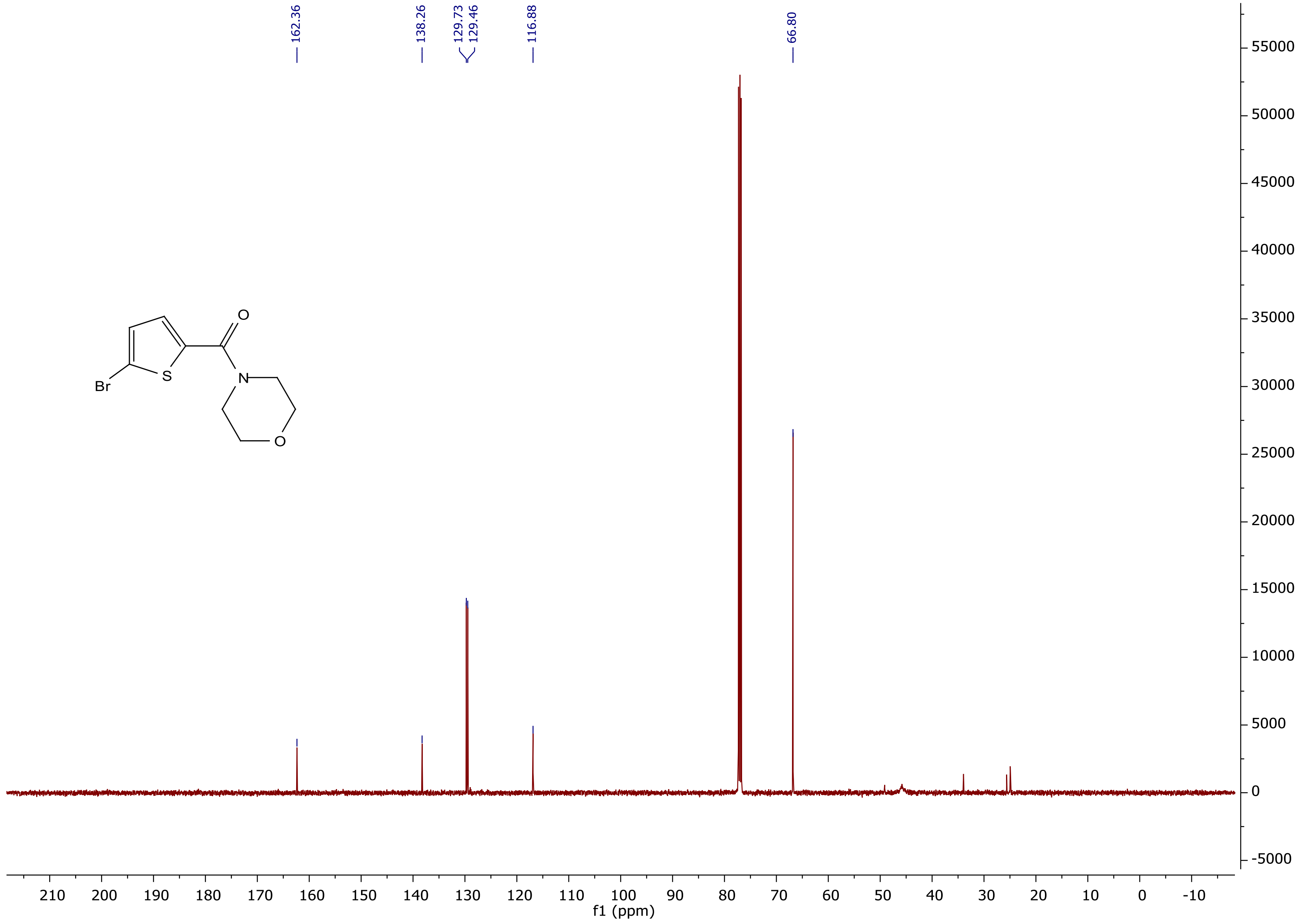


**Figure S17**. $^{13}$C-NMR of **MB-D3** in $CDCl_3$, (125 MHz), 298K

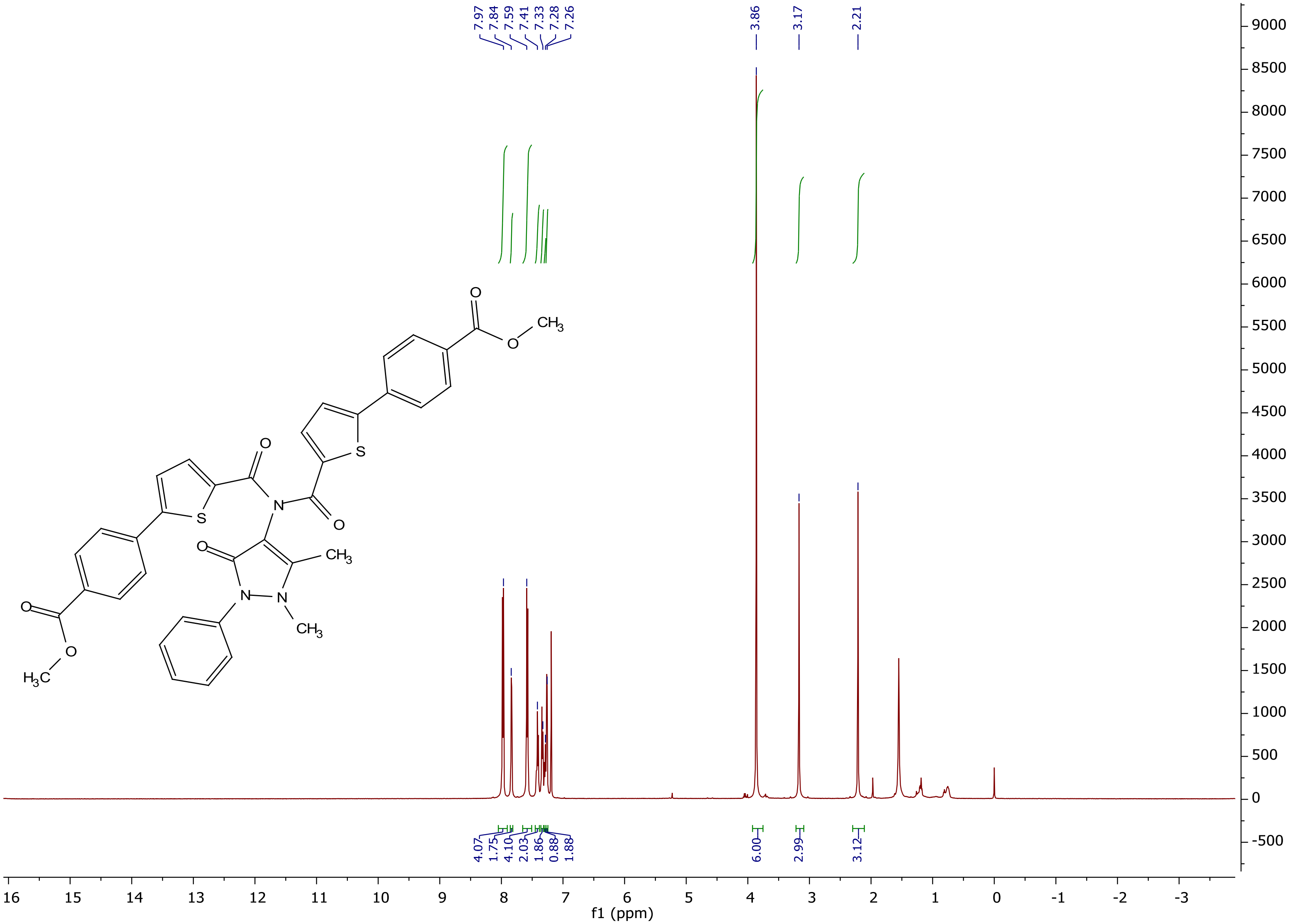


**Figure S18**. $^{1}$H-NMR spectrum of **MB-D4** in $CDCl_3$, (500 MHz), 298K

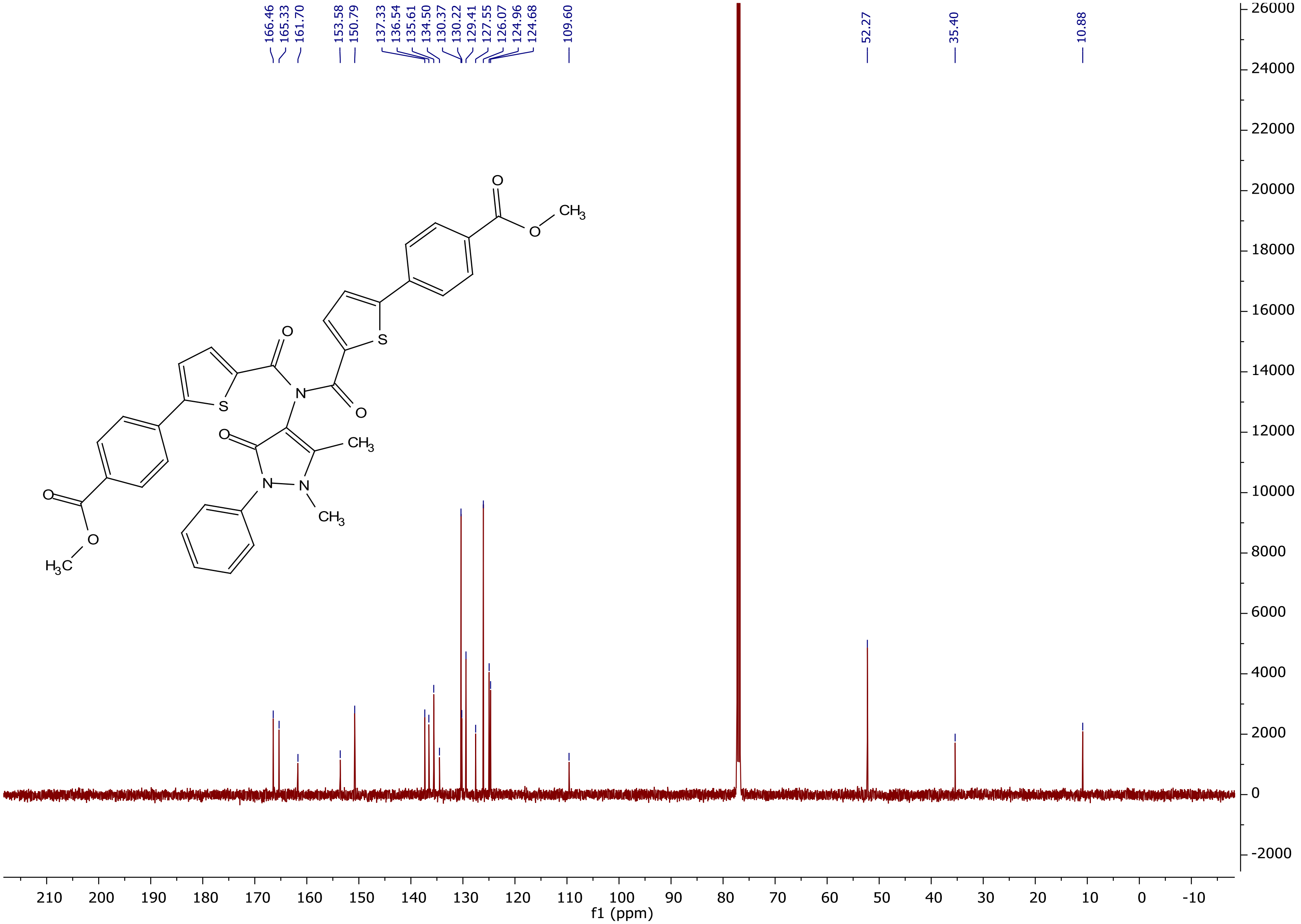


**Figure S19**. $^{13}$C-NMR of **MB-D4** in $CDCl_3$, (125 MHz), 298K

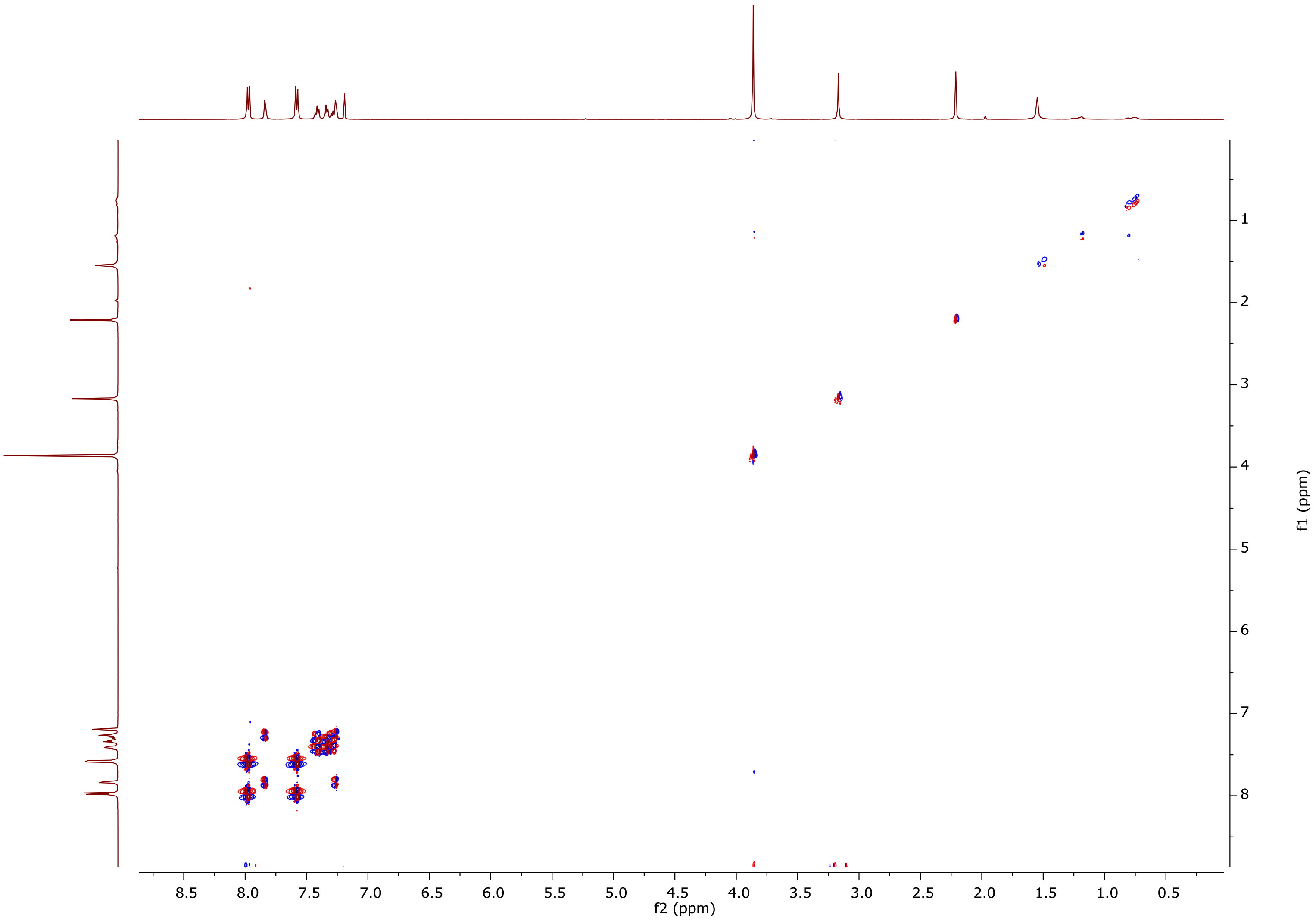


**Figure S20**. $^{1}H$-$^{1}H$-COSY of **MB-D4** in $CDCl_3$, (125 MHz), 298K

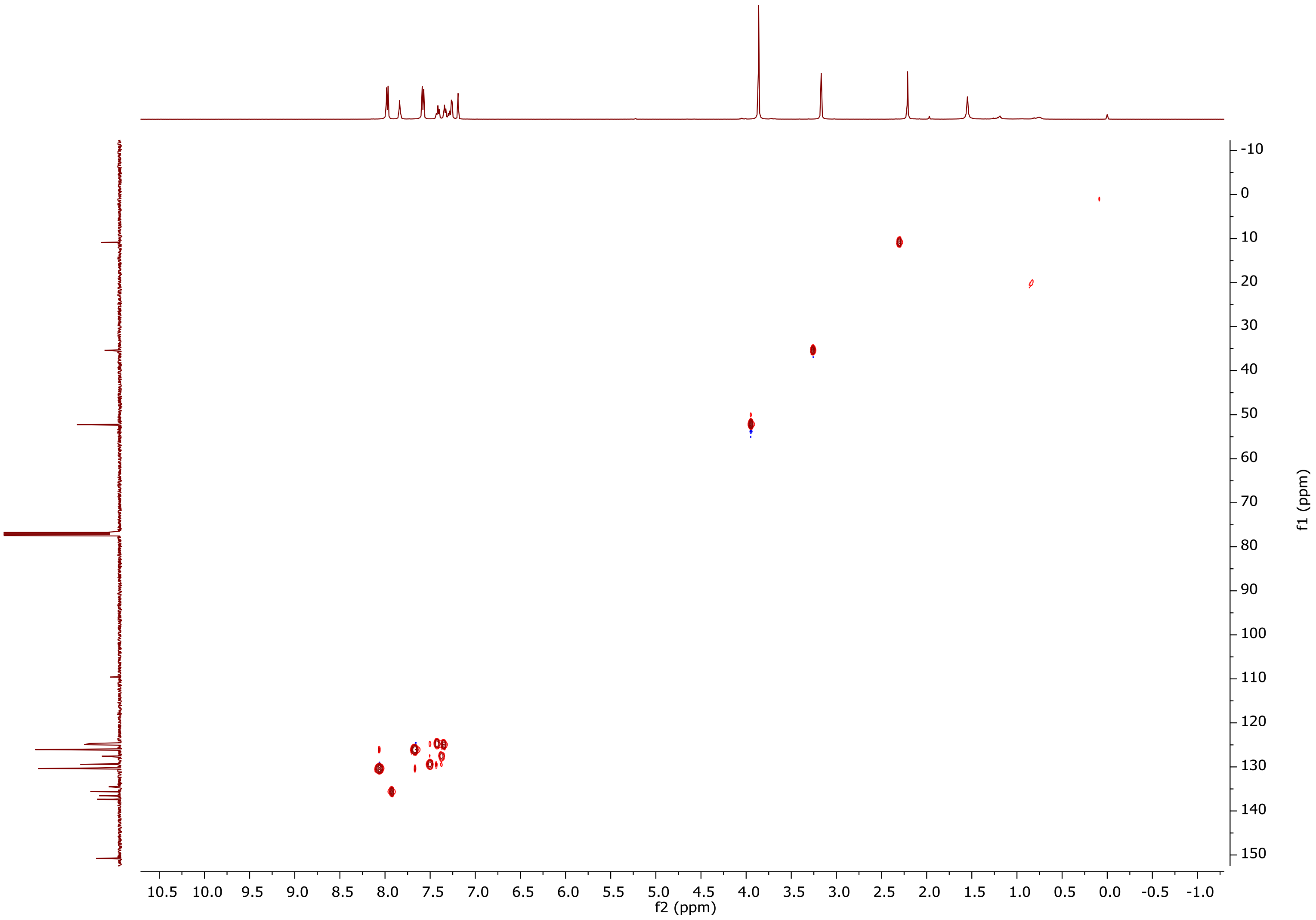


**Figure S21**. $^1$H-$^{13}$C-HSQC of **MB-D4** in $CDCl_3$, (125 MHz), 298K

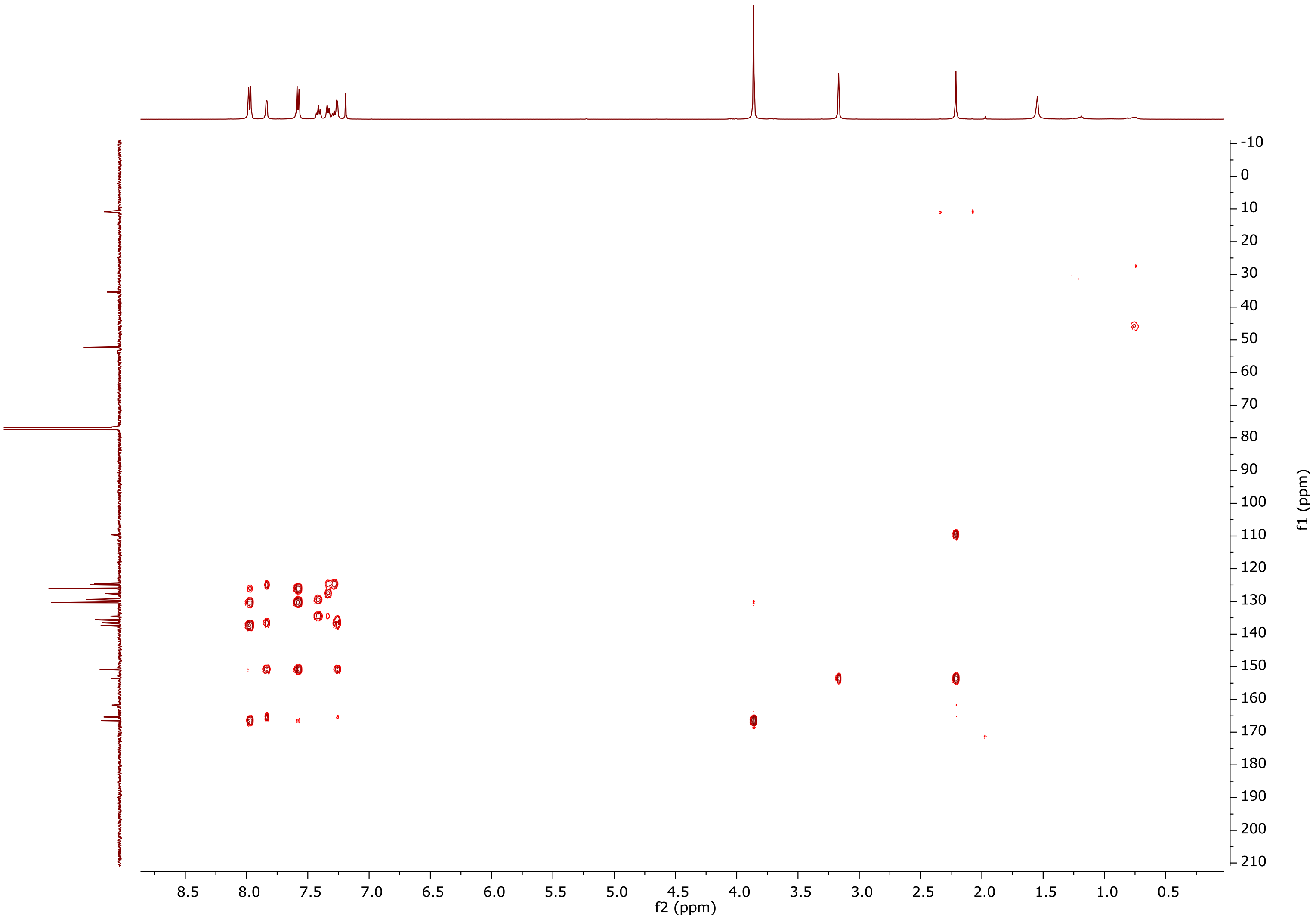


**Figure S22**. $^{1}H$-$^{13}C$-HMBC of **MB-D4** in $CDCl_3$, (125 MHz), 298K

**Table S1.** MS source and acquisition parameters in positive mode

| Source parameters | Values |
|---|---|
| Ionization mode | Positive electrospray ionization |
| Spray voltage | 3500 - 4800 V |
| Sheath gas flow rate | 5 Arb |
| Auxiliary gas flow rate | 2 Arb |
| Sweep gas flow rate | 0 Arb |
| Ion transfer tube temperature | 300℃ |
| Vaporizer temperature | 20℃ |
| **Acquisition parameters** | **Values** |
| Detector type | Orbitrap |
| Orbitrap resolution | 120000 |
| Scan range | 150-1000 m/z |
| AGC target | 100 % |
| Injection time | 100 ms |
| Syringe flow | 5 µl/min |

**Tabel S2**. 1D and 2D -NMR full peak assignment of **MB-D2**

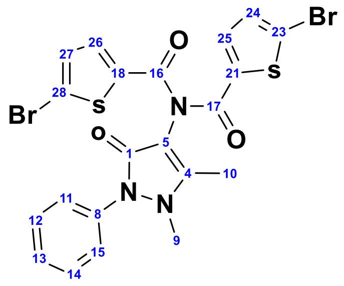


| Position | $^{1}H$ δ/ppm (J/Hz) | $^{13}C$ δ/ppm | HMBC |
|---|---|---|---|
| 25;26 | 7.61 (d, J = 4.1 Hz, 2H) | 135.2 | H24, H27 |
| 12;14 | 7.41 (m, 2H) | 129.5 | H12, H14 |
| 11;15 | 7.31 (d, J = 2.2 Hz, 2H) | 125.1 | H12, H13, H14 |
| 13 | 7.29 (m, 1H) | 127.8 | H11, H15 |
| 24;27 | 6.96 (d, J = 4.1 Hz, 2H) | 131.0 | H25, H26 |
| 9 | 3.18 (s, 3H) | 35.2 | - |
| 10 | 2.18 (s, 3H) | 10.8 | - |
| 16;17 | - | 164.3 | H10, H24, H25, H26, H27 |
| 1 | - | 161.4 | H10 |
| 4 | - | 153.2 | H9, H10 |
| 18;21 | - | 137.4 | H24, H25, H26, H27 |
| 8 | - | 134.1 | H12, H14 |
| 23;28 | - | 122.2 | H24, H25, H26, H27 |
| 5 | - | 108.44 | H10 |

**Tabel S3**. 1D and 2D -NMR full peak assignment of **MB-D4**

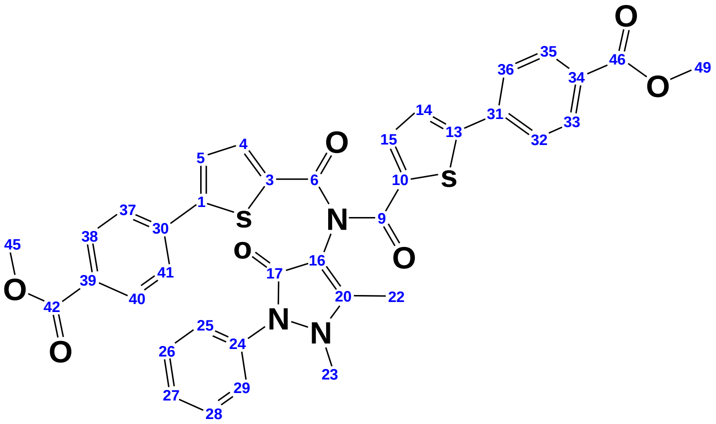

| **Position** | **$^{1}$H δ/ppm (J/Hz)** | **$^{13}$C δ/ppm** | **HMBC** |
|---|---|---|---|
| 35;33;40;38 | 7.97 (m, 4H) | 130.4 | H35, H33, H40, H38, H41, H37, H36, H32 |
| 4;15 | 7.84 (d, J= 4.0 Hz, 2H) | 135.6 | H5, H14 |
| 36;32;41;37 | 7.59 (d, J = 8.4 Hz, 4H) | 126.1 | H33, H35, H40, H38, H36, H32, H41, H37 |
| 26;28 | 7.41 (t, J = 7.6 Hz, 2H) | 129.4 | H26, H28, H32, H33, H35, H36, H37, H38, H40, H41 |
| 25;29 | 7.33 (d, J = 7.8 Hz, 2H) | 124.7 | H4, H15, H5, H14, H25, H27, H29 |
| 27 | 7.28 (d, J = 7.4 Hz, 1H) | 127.6 | H25,H29 |
| 5;14 | 7.26 (d, J = 4.1 Hz, 2H) | 125.0 | H4, H15, H25, H27, H29 |
| 45;49 | 3.86 (s, 6H) | 52.3 | - |
| 23 | 3.17 (s, 3H) | 35.4 | - |
| 22 | 2.21 (s, 3H) | 10.9 | - |
| 42;46 | - | 166.5 | H49, H45, H40, H38, H35, H33, H41, H37, H36, H32 |

| | | | |
|---|---|---|---|
| 6;9 | - | 165.3 | H4, H15 |
| 17 | - | 161.7 | - |
| 20 | - | 153.6 | H23 |
| 1;13 | - | 150.8 | H4, H5, H14, H15, H32, H36, H37, H41 |
| 30;31 | - | 137.3 | H4, H5, H14, H15, H33, H35, H38, H40 |
| 3;10 | - | 136.5 | H4, H5, H14, H15 |
| 4;15 | - | 135.6 | H5, H14 |
| 24 | - | 134.5 | H26, H28 |
| 35;33;40;38 | - | 130.4 | H35, H33, H40, H38, H41, H37, H36, H32 |
| 34;39 | - | 130.2 | H32, H33, H35, H36, H37, H38, H40, H41 |
| 16 | - | 109.6 | H22 |
